# TRAK adaptors regulate the recruitment and activation of dynein and kinesin in mitochondrial transport

**DOI:** 10.1038/s41467-023-36945-8

**Published:** 2023-03-13

**Authors:** John T. Canty, Andrew Hensley, Merve Aslan, Amanda Jack, Ahmet Yildiz

**Affiliations:** 1 Biophysics Graduate Group, University of California at Berkeley, Berkeley, CA 94720 USA; 2 Physics Department, University of California at Berkeley, Berkeley, CA 94720 USA; 3 Department of Molecular and Cellular Biology, University of California at Berkeley, Berkeley, CA 94720 USA; 4 Present Address: Department of Cancer Immunology, Genentech Inc., 1 DNA Way, 94080 South San Francisco, CA USA

**Keywords:** Motor protein regulation, Single-molecule biophysics, Mitochondria, Microtubules

## Abstract

Mitochondrial transport along microtubules is mediated by Miro1 and TRAK adaptors that recruit kinesin-1 and dynein-dynactin. To understand how these opposing motors are regulated during mitochondrial transport, we reconstitute the bidirectional transport of Miro1/TRAK along microtubules in vitro. We show that the coiled-coil domain of TRAK activates dynein-dynactin and enhances the motility of kinesin-1 activated by its cofactor MAP7. We find that TRAK adaptors that recruit both motors move towards kinesin-1’s direction, whereas kinesin-1 is excluded from binding TRAK transported by dynein-dynactin, avoiding motor tug-of-war. We also test the predictions of the models that explain how mitochondrial transport stalls in regions with elevated Ca^2+^. Transport of Miro1/TRAK by kinesin-1 is not affected by Ca^2+^. Instead, we demonstrate that the microtubule docking protein syntaphilin induces resistive forces that stall kinesin-1 and dynein-driven motility. Our results suggest that mitochondrial transport stalls by Ca^2+^-mediated recruitment of syntaphilin to the mitochondrial membrane, not by disruption of the transport machinery.

## Abstract (web summary)

The mechanisms of microtubule-based mitochondrial transport remain poorly understood. Here, the authors show that the mitochondrial TRAK adaptors activate the dynein-dynactin complex, enhance the motility of kinesin, and can scaffold both motors to control bidirectional transport.

## Introduction

Mitochondria are cellular power plants that generate most of the ATP needed for many biochemical reactions and have a high capacity to buffer cytosolic Ca^2+^. In neurons, mitochondria are distributed to distal regions where ATP and Ca^2+^ buffering are in high demand, such as synapses and axonal branches^1^. Mitochondrial transport is essential for axonal growth and branching, maintaining action potentials, and supporting synaptic transmission^1^. Aged and dysfunctional mitochondria need to be transported back to the cell body for degradation^2^. Defects in mitochondrial transport are associated with a variety of neurodegenerative disorders, including Parkinson’s disease^1^.

Early genetic screens identified the mitochondrial Rho GTPases Miro1 and Miro2 as essential factors regulating mitochondrial transport and quality control^3^. Miro1 is composed of two GTPase domains and two EF-hands that bind Ca^2+^. Miro1 is localized to the outer mitochondrial membrane through its transmembrane domain^4,5^, and is coupled to the C-terminus of the trafficking of kinesin-binding (TRAK) adaptors^6^. The N-terminal coiled-coil domain of TRAK1 and TRAK2 recruit kinesin-1 and dynein-dynactin^4,7^, which transport mitochondria towards the plus- and minus-ends of microtubules (MTs), respectively^7–9^. Co-immunoprecipitation studies showed that TRAK1 recruits both dynein and kinesin whereas TRAK2 primarily interacts with dynein^7^. TRAK1 was found to be enriched in the axons of cultured neurons, while TRAK2 was found mainly in dendrites^7^, suggesting that these adaptors have nonredundant roles in mitochondrial trafficking.

Live cell imaging studies showed that mitochondria exhibit rapid anterograde and retrograde transport, interspersed with infrequent pausing and directional switching in axons and dendrites^10–13^. The complex transport properties of mitochondria are primarily driven by Miro1, TRAK adaptors, motors, and other associated factors^1^, but little is known about how these components control directionality and pausing of mitochondria. In vitro reconstitution studies showed that TRAK1 binds and activates kinesin motility^14^. TRAK2 was also shown to recruit both kinesin and dynein and regulate their activity in cell extracts^15^, but the underlying mechanism of how TRAK facilitates the activation and coordination of these opposing motors and mediates the transport of Miro1 is not well understood.

In mature neurons, two-thirds of mitochondria are docked to MTs^1^. Studies in cultured neurons demonstrated that mitochondrial transport stalls when local Ca^2+^ concentrations are elevated^8^. Yet a rise in the cytosolic Ca^2+^ concentration fails to halt mitochondrial transport when an EF-hand mutant of Miro1 is expressed in neurons and other cell types^5,16,17^, indicating that Miro is the Ca^2+^ sensor that facilitates this process. However, the mechanism by which Ca^2+^ binding to Miro1 prevents motors from driving mitochondrial transport remains controversial^5,17,18^. The ‘motor detachment’ model proposes that Ca^2+^ binding to Miro1 decouples kinesin from TRAK^5^. In the ‘Miro-binding’ model, Ca^2+^ binding causes Miro1 to directly interact with the kinesin motor domain and inhibits its motility^17^. These models do not explain how elevated Ca^2+^ concentration also stops the retrograde mitochondrial transport driven by dynein. In addition, the knockdown of both Miro1 and Miro2 does not completely suppress Ca^2+^-induced arrest of mitochondria in neurons^16,19^, suggesting that the Ca^2+^-mediated arrest may function independently of the transport machinery. Recent studies in mouse models proposed an alternative model, in which a mitochondrial docking protein, syntaphilin (SNPH) anchors mitochondria to MTs in axons^18^. SNPH is recruited to mitochondria in response to sustained neuronal activity and elevated Ca^2+^ levels^18^. This model is supported by the observations that overexpression of SNPH completely abolishes mitochondrial transport in both directions and that increasing cytosolic Ca^2+^ fails to arrest mitochondrial transport in axons of SNPH knock-out neurons^18,20^. The “engine-switch and brake” model proposes that SNPH inhibits kinesin through direct molecular interactions and serves as a brake by anchoring mitochondria to MTs^20^. The predictions of these models could not be directly tested in vitro due to the lack of reconstituted assays from purified components.

In this study, we investigated the role of Miro1 and TRAK in the recruitment and activation of dynein and kinesin motility using in vitro reconstitution. We show that TRAK1 and TRAK2 activate dynein-dynactin motility. TRAK1 also increases the kinesin landing rate onto MTs but the MT-associated protein MAP7 is required to stimulate robust kinesin motility. TRAK1 or TRAK2 can simultaneously recruit dynein-dynactin and kinesin and these complexes are exclusively transported to the plus-end of MTs by kinesin. In comparison, kinesin does not colocalize to the TRAK adaptors transported to the minus-end by dynein/dynactin, demonstrating that TRAK coordinates the activity of opposing motors to avoid futile tug-of-war. We also distinguished between the predictions of the existing models of Ca^2+^-mediated stalling of mitochondrial transport. Miro1 stably interacts with kinesin/TRAK, and the motility of this complex is unaffected by excess Ca^2+^. However, static anchoring by SNPH is sufficient to stall kinesin or dynein motility. These results provide insight into the regulation of mitochondrial transport.

## Results

### TRAK1 and TRAK2 are activating adaptors of dynein-dynactin

**Fig. 1:**
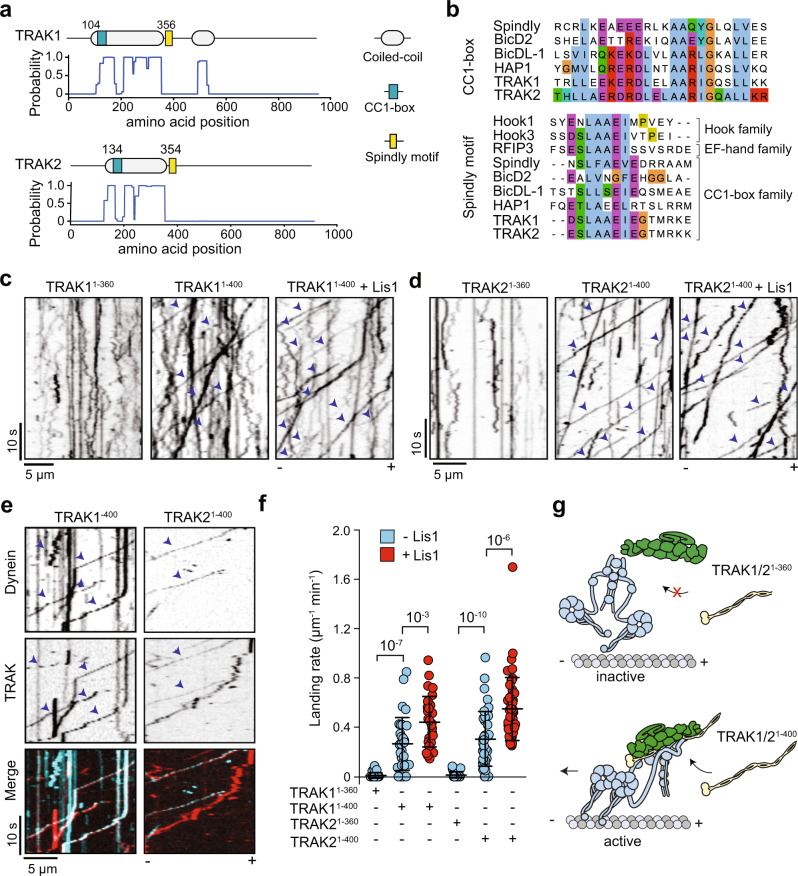
TRAK coiled-coil domains activate dynein-dynactin motility. **a** Domain organization and coiled-coil prediction score of human TRAK1 and TRAK2. **b** Sequence alignment shows the conserved CC1 box (top) and the Spindly motif (bottom) of TRAK1 and TRAK2 with other activating adaptors of human dynein-1. The sequences were aligned using the Clustal Omega algorithm. **c**, **d** Representative kymographs of TRAK1 (**c**) and TRAK2 (**d**) constructs labeled with the LD655 dye in the presence of unlabeled dynein/dynactin (DD) with or without 1 µM Lis1. Arrowheads highlight processive motility. **e** Representative kymographs of LD655-dynein and LD555-TRAK constructs in the presence of unlabeled dynactin. Arrowheads represent TRAK-dynein colocalization. The processive motility of TRAK not colocalizing with dynein is due to less than 100% labeling efficiency of dynein. **f** The landing rate of motor complexes on MTs. The centerline and whiskers represent mean and s.d., respectively (*n =* 30, 32, 31, 31, 44, and 48 MTs from left to right). *P* values are calculated from a two-tailed *t*-test. **g** TRAK is an activating adaptor of dynein-dynactin. Activation of dynein motility requires both the CC1 box and the Spindly motif in the TRAK coiled-coil.

Recent studies have identified a family of coiled-coil adaptor proteins that activate dynein motility by recruiting one or two dynein motors to dynactin^21,22^. Sequence alignments with established dynein adaptors confirmed that TRAK1 and TRAK2 contain the CC1 box that binds the dynein light-intermediate chain (LIC)^21,23^ and the Spindly motif that interacts with the pointed-end of dynactin^24^ (Fig. 1a,b). We first investigated whether human TRAK1/2 could activate mammalian dynein-dynactin for processive motility using single-molecule imaging in vitro (Supplementary Fig. 1a). In the absence of TRAK, dynein-dynactin exhibited little to no motility, as previously shown^25,26^ **(**Supplementary Fig. 1b). Remarkably, the N-terminal coiled coils of TRAK1/2 that contain both the CC1 box and the Spindly motif (TRAK1^1–400^ and TRAK2^1–400^) led to robust activation of dynein-dynactin motility towards the MT minus-end **(**Fig. 1c,d, Supplementary Fig. 1b, and Supplementary Video 1). The velocities of dynein-dynactin-TRAK1^1–400^ (DDT_1_^1–400^), and -TRAK2^1–400^ (DDT_2_^1–400^) complexes (810 ± 20 and 870 ± 20 nm s^−1^, mean ± s.e.m., respectively) were comparable to that of dynein-dynactin assembled with BicD adaptors in vitro^25–27^ and the retrograde transport speed of mitochondria (300–900 nm s^−1^) in vivo^7,12^ (Supplementary Fig. 1c). In comparison, TRAK1/2 constructs that contain the CC1 box but lack the Spindly motif (TRAK1^1–360^ and TRAK2^1–360^) resulted in only occasional motility (Fig. 1c,d), underscoring the importance of the Spindly motif in the activation of dynein-dynactin.

Multi-color tracking experiments visualized direct colocalization of motile dynein and TRAK adaptors (Fig. 1e,f), demonstrating that TRAK needs to be part of the dynein-dynactin complex to sustain processive motility. The MT landing rate of active DDT_1_^1–400^ and DDT_2_^1–400^ complexes increased two-fold with the addition of 1 µM Lis1 (Fig. 1c,d,f and Supplementary Fig 1b), a dynein regulatory protein that facilitates the assembly of active dynein-dynactin-adaptor complexes^28–30^. Collectively, our results showed that TRAK1 and TRAK2 are activating adaptors of dynein-dynactin for mitochondrial transport (Fig. 1g).

### TRAK binding increases the frequency of kinesin motility

**Fig. 2:**
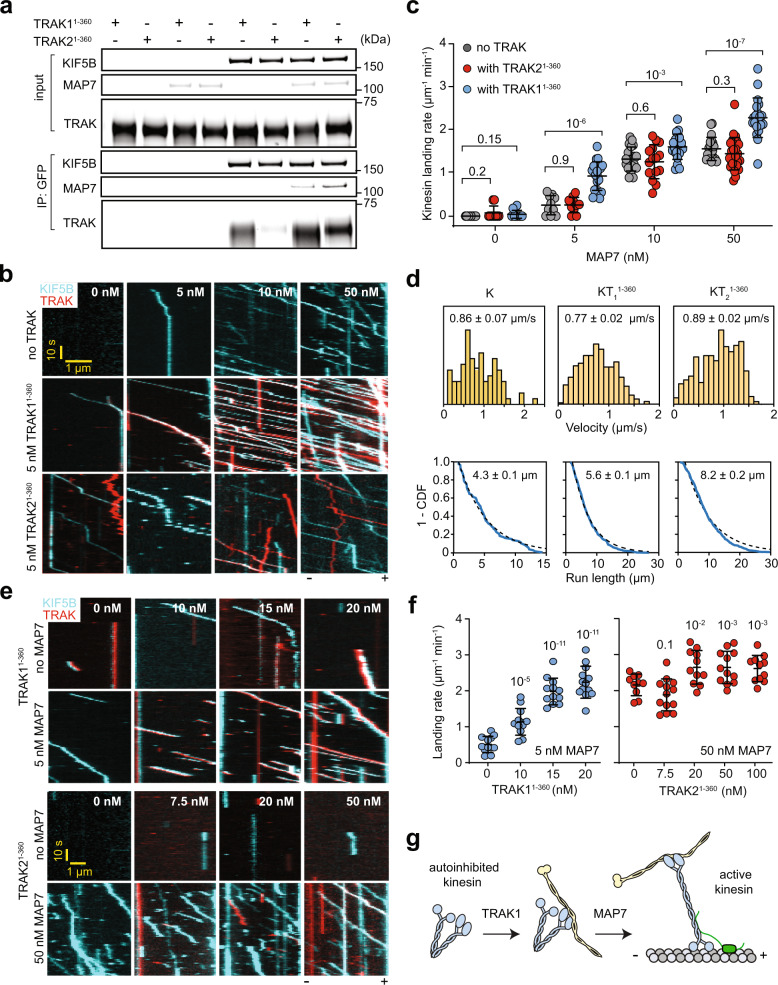
Kinesin recruits TRAK adaptors more efficiently following activation by MAP7. **a** In vitro immunoprecipitation (IP) of purified kinesin (KIF5B-GFP-SNAPf), TRAK1^1–360^, and TRAK2^1–360^ in the presence or absence of 10 nM MAP7. The proteins were eluted from anti-GFP beads. **b** Representative two-color kymographs of KIF5B and TRAK constructs with increasing concentrations of MAP7. The processive motility of TRAK not colocalizing with kinesin is due to less than 100% labeling of kinesin. **c**, The landing rate of kinesin in the absence and presence of 5 nM TRAK1^1–360^ or TRAK2^1–360^ under increasing MAP7 concentrations a(*n* = 10, 10, 10, 10, 10, 20, 20, 15, 20, 20, 25, and 20 MTs from left to right, two independent trials). **d** (Top) Velocity histogram (mean ± s.e.m.) and (Bottom) the inverse cumulative distribution function (1-CDF) of motor run length for K (*n* = 88), KT_1_^1–360^ (*n* = 404), and KT_2_^1–360^ (*n* = 499, three independent experiments) in 10 nM MAP7. Fits to a single exponential decay (dashed curves) reveal the motor run length (±s.e.). **e** Representative two-color kymographs of LD555-kinesin with increasing concentrations of LD655-labeled TRAK1^1–360^ or TRAK2^1–360^ in the presence or absence of MAP7. A higher MAP7 concentration (50 nM) was used for TRAK2 because kinesin runs remained infrequent at a lower MAP7 concentration (5 nM). **f** The landing rate of kinesin with increasing concentrations of TRAK adaptors. The centerline and whiskers represent mean and s.d., respectively (*n* = 12, 12, 12, 13, 10, 13, 11, 12, and 11 MTs from left to right, three independent trials). **g** TRAK recruits kinesin, but activation of KT motility requires a kinesin-1 cofactor, MAP7. In (**c**) and (**f**), the center line and whiskers represent the mean and s.d., respectively. P-values are calculated from a two-tailed t-test. In (**f**), *p* values are calculated in comparison to the no TRAK condition.

A previous study reported that the N-terminal coiled-coil domain of TRAK recruits the kinesin heavy chain^7^. Consistent with this report, our in vitro pull-down assays using purified proteins revealed that full-length human kinesin-1 heavy chain (KIF5B, kinesin hereafter) binds to TRAK1^1–360^, and to a lesser extent TRAK2^1–360^ (Fig. 2a, Supplementary Fig. 2a). To determine how TRAK binding affects the activation and motility of kinesin, we performed single-molecule motility assays of kinesin with and without TRAK adaptors in vitro (Fig. 2b-d). In the absence of TRAK, kinesin landed infrequently onto the MTs and did not walk along the MT (Fig. 2b,c), consistent with autoinhibition of this motor^31^. In the presence of 5 nM TRAK1^1–360^ or TRAK2^1–360^, we only observed occasional motility of kinesin-TRAK (KT) complexes on MTs (Fig. 2b,c). A previous study^14^ reported activation of kinesin motility by a higher concentration (100-150 nM) of full-length TRAK1, but we observed little to no increase in kinesin landing rate by the addition of 20-100 nM TRAK1^1–360^, suggesting that TRAK alone is insufficient to trigger robust activation of kinesin motility (Fig. 2e).

We next asked whether TRAK adaptors more efficiently recruit kinesin when this motor is activated by its required cofactor, MAP7^32–34^. In vitro pull-down assays showed that kinesin binds more strongly to TRAK1^1–360^ and TRAK2^1–360^ in the presence of 10 nM MAP7 (Fig. 2a). Consistent with previous reports^32–34^, the landing rate and mobile fraction of kinesin increased 10-40 fold when we decorated MTs using 5 – 50 nM MAP7 in motility assays (Fig. 2b,c and Supplementary Video 2). Under the same MAP7 concentration, the addition of 5 nM TRAK1^1–360^ increased the landing rate of kinesin- TRAK1^1–360^ complexes (KT_1_^1–360^) about 1.5-fold compared to the no TRAK condition, while we did not observe a significant increase in the presence of 5 nM TRAK2^1–360^ (Fig. 2b,c). Similarly, the landing rate and percent colocalization of TRAK1^1–360^ and TRAK2^1–360^ adaptors to processive kinesins increased under higher MAP7 concentrations, with TRAK1^1–360^ colocalizing with kinesin more efficiently than TRAK2^1–360^ (Supplementary Fig. 2b). We also observed the comigration of longer (TRAK1^1–400^ and TRAK2^1–400^) and full-length (TRAK1^1–953^ and TRAK2^1–914^) TRAK constructs with kinesin on MTs (Supplementary Fig. 2c-e). However, the landing rate of KT complexes assembled with full-length TRAK was noticeably infrequent than those formed by shorter TRAK constructs that lack its C-terminus, consistent with a previous report that the C-terminus of TRAK reduces the affinity between the N-terminal coiled-coil and kinesin^7^.

We also tested whether an increase in TRAK concentration can lead to more robust kinesin motility in the presence of MAP7. Compared to the condition without TRAK, the addition of 20 nM TRAK1^1–360^ increased the kinesin landing rate by 5-fold, whereas the addition of 100 nM TRAK2^1–360^ resulted in a modest increase (30%) (Fig. 2e,f). Collectively, our results show that the coiled-coil domain of TRAK1, and to a lesser extent TRAK2, efficiently recruits kinesin when the motor is rescued from autoinhibition by MAP7 (Fig. 2g).

Kinesin motors co-localized with TRAK adaptors moved at similar velocities but exhibited higher run lengths than kinesin alone (Fig. 2d). A previous study also reported longer run lengths for kinesin transporting full-length TRAK1 and attributed this increase in processivity to the interaction of the TRAK1 C-terminus with MTs^14^. We also observed MT binding of 150 nM TRAK1^1–360^ and a TRAK1 construct containing the coiled coils and part of the C-terminal domain (TRAK1^1–532^) in the absence of kinesin. However, the affinity of TRAK for MTs was weak and we could not detect MT binding of TRAK1^1–360^, TRAK1^1–532^, or full-length TRAK1 when TRAK concentration was lowered to 20 nM (Supplementary Fig. 3). Because kinesin run length increases when it transports a TRAK construct that lacks its C-terminus (TRAK1^1–360^) at low TRAK concentrations (Fig. 2d), we concluded that KT processivity increases even without MT binding of the TRAK C-terminus.

### Force generation of complexes formed with TRAK adaptors

**Fig. 3:**
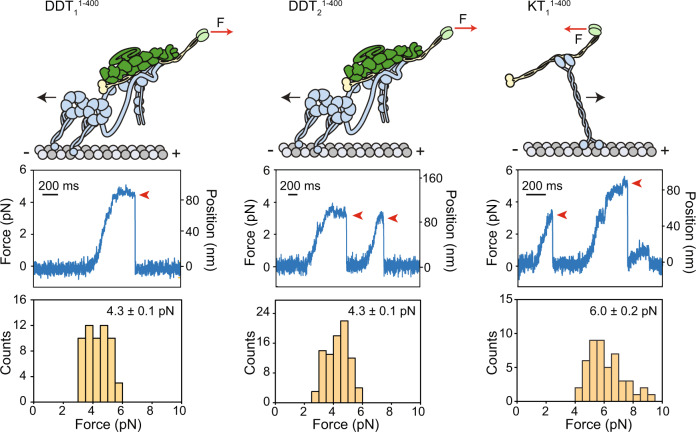
Force generation of dynein-dynactin or kinesin assembled with TRAK adaptors. (Top) Single motor complexes were pulled from the TRAK adaptor by an optically trapped bead (not shown; F: force). (Middle) Representative traces of beads driven by a single KT or DDT complex in a fixed-trap assay. Red arrowheads represent the detachment of the motor from an MT after the stall. (Bottom) Stall force histograms (mean ± s.e.m.) of DDT_1_^1–400^ (*n* = 62 stalls from 15 beads in 7 independent experiments), DDT_2_^1–400^ (*n* = 86 stalls from 14 beads in 6 independent experiments) and KT_1_^1–400^ (*n* = 55 stalls from 14 beads in 6 independent experiments).

To test how TRAK binding affects the force production of dynein and kinesin, we measured the stall forces of KT_1_^1–360^, DDT_1_^1–400^, and DDT_2_^1–400^ complexes using an optical trap (Supplementary Fig. 4a,b). To ensure that the forces measured corresponded to a fully assembled complex, we attached the beads directly to TRAK adaptors using a GFP-antibody linkage^35^ (Fig. 3). Both DDT_1_^1–400^ and DDT_2_^1–400^ complexes stalled when subjected to 4.3 pN resistive forces (Fig. 3) and exhibited similar stall times before MT detachment (Supplementary Fig. 4c). These forces are comparable to that of complexes that contain a single dynein motor and are lower than the complexes that contain two dyneins^35,36^, suggesting that TRAK primarily recruits one dynein to dynactin in our reconstitution conditions. KT_1_^1–360^ stalled at 5.96 ± 0.24 pN load (Fig. 3), which closely matched to the stall force of constitutively active kinesin^37^, indicating that TRAK recruits a single kinesin motor in our reconstitution conditions^14^. We were unable to detect any bead motility in the presence of TRAK2^1–360^, consistent with the low affinity of kinesin for this construct. These results show that DDT and KT are active complexes that generate sufficient force to drive retrograde and anterograde motility.

### TRAK simultaneously recruits dynein-dynactin and kinesin

**Fig. 4:**
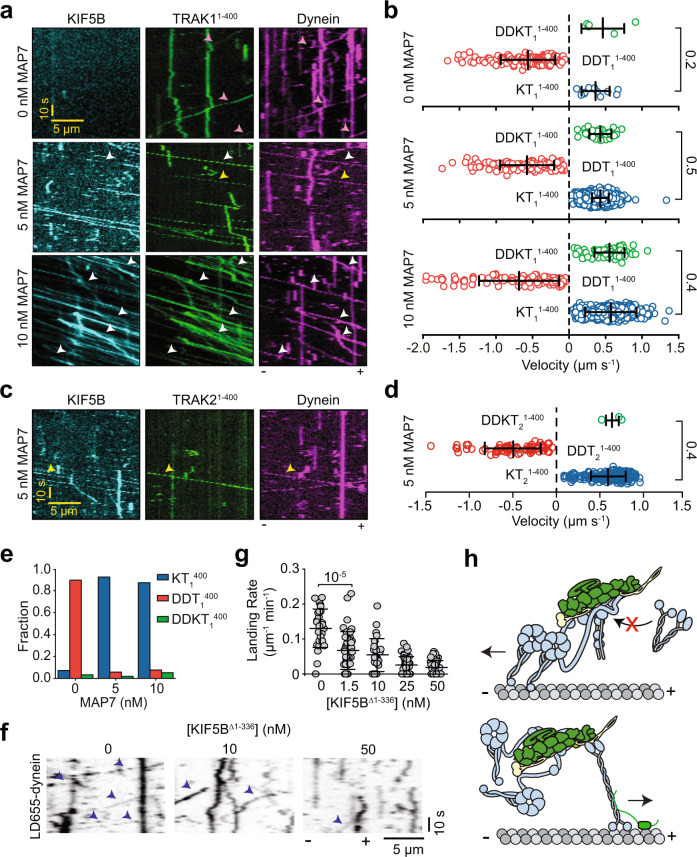
TRAK adaptors simultaneously recruit dynein-dynactin and kinesin. **a** Representative kymographs of Alexa488-kinesin, LD555-TRAK1^1–400^, and LD655-dynein in the presence of 0, 5, and 10 nM MAP7. White arrowheads show colocalization of dynein, kinesin, and TRAK. Yellow arrowheads highlight the plus-end-directed movement of dynein and TRAK by unlabeled kinesin. Magenta arrowheads represent the minus-end-directed movement of dynein and TRAK. **b** The velocity distribution of complex assemblies at 0, 5, and 10 nM MAP7. Negative velocities correspond to minus-end-directed motility. (*n* = 5, 138, 11, 43, 130, 2104, 79, 117, and 1342 from top to bottom, three independent experiments per condition). **c** Representative kymographs of Alexa488-kinesin, LD555-TRAK2^1–400^, and LD655-dynein on a surface-immobilized MT in the presence of 5 nM MAP7. Assays were performed in the absence of Lis1. Yellow arrowheads show colocalization of dynein, kinesin, and TRAK2. **d** The velocity distribution of complex assemblies at 5 nM MAP7 and 0 nM Lis1 (*n =* 3, 67, and 206 from top to bottom, three independent experiments per condition). **e** The fraction of complexes formed with TRAK_1_^1–400^ under different MAP7 concentrations. **f** Sample kymographs of 0.5 nM DDT1^1–400^ assemblies in the presence of 1 µM Lis1 and increasing concentrations of KIF5B^Δ1-336^. Processive runs of LD655-dynein are highlighted with blue arrowheads. **g** The landing rates of 0.5 nM DDT_1_^1–400^ assemblies under increasing concentrations of KIF5B^Δ1-336^ (*n* = 28, 45, 27, 50, and 51 MTs from left to right; two independent experiments per condition). **h** Schematic representation of motor coordination on TRAK. When kinesin transports TRAK, dynein can remain as an inactive passenger, but kinesin is excluded from minus-end directed DDT complexes. In (**b**), (**d**), and (**g**), the center line and whiskers represent the mean and s.d., respectively. P-values are calculated from a two-tailed t-test.

To test whether dynein and kinesin can colocalize to the same TRAK adaptor, we performed three-color imaging of dynein-dynactin, kinesin, and either TRAK1^1–400^ or TRAK2^1–400^. We observed dynein-dynactin/kinesin/TRAK1^1–400^ (DDKT_1_^1–400^) colocalizers moving along the MT (Fig. 4a,b and Supplementary Video 3). The likelihood of detecting dynein and kinesin to simultaneously colocalize on TRAK2^1–400^ was substantially lower, presumably because kinesin has a low affinity for TRAK2 (Fig. 4c,d). The analysis of these trajectories revealed that all DDKT_1_^1–400^ and DDKT_2_^1–400^ assemblies moved towards the MT plus-end at similar velocities to KT_1_^1–400^ and KT_2_^1–400^ complexes in the same chamber (Figs. 4b, d, and Supplementary Fig. 5b). The velocity of DDKT_1_^1–400^ and DDKT_2_^1–400^ complexes were substantially higher than the case in which kinesin and dynein engage in a tug-of-war on an artificial DNA scaffold^27,35,38^, indicating that dynein is not competing against kinesin-driven motility when both motors are recruited by TRAK.

We next investigated why DDKT complexes exclusively move towards the plus-end. We first tested different concentrations of MAP7, which activates full-length kinesin, while reducing the landing rate of dynein motility^34^. Without MAP7, we predominantly observed DDT motility, and a small number of KT or DDKT complexes on MTs when we used either TRAK1^1–400^ (Fig. 4a,b) or TRAK2^1–400^ (Supplementary Fig. 5a,b). In contrast, at 5 and 10 nM MAP7, we mostly observed processive runs of KT complexes on MTs (Fig. 4e and Supplementary Fig. 5b) and a slight reduction in the landing rate of active DDT complexes. Importantly, in all MAP7 concentrations we tested, both DDKT_1_^1–400^ and DDKT_2_^1–400^ complexes always moved toward the plus-end (Fig. 4b). Thus, the concentration of MAP7 acts to tune bidirectional motility by activating kinesin and interfering with dynein motility, but it does not affect the directionality of DDKT complexes. We also tested whether dynein more efficiently competes against kinesin when its assembly with dynactin and TRAK is aided by Lis1^28–30^. The addition of 1 μM Lis1 increased the landing rate of both DDT_1_^1–400^ and DDT_2_^1–400^ without affecting kinesin motility. However, the Lis1 addition had only a minor effect on the landing rate of complexes containing both motors, and no effect on their directional preference (Supplementary Fig. 5c,d), ruling out this possibility.

We also considered the possibility that the direction of DDKT motility is driven by higher force generation of kinesin compared to single dynein (Fig. 3). To test this possibility, we replaced full-length kinesin with a kinesin tail construct that binds to TRAK but lacks the motor domain needed for plus-end directed motility and force generation (KIF5B^Δ1–336^). If the direction of DDKT is determined by mechanical competition, we expected DDKT_1_^1–400^ complexes assembled with KIF5B^Δ1–336^ to move towards the minus end. However, the addition of excess KIF5B^Δ1–336^ only resulted in more than a 5-fold reduction in DDT_1_^1–400^ landing rate (Fig. 4f,g), but we did not observe any KIF5B^Δ1-336^ colocalizing with processive DDT_1_^1–400^ complexes moving towards the minus end. These results indicate that when kinesin transports TRAK, dynein-dynactin cannot form an active motor, but it can be transported by kinesin towards the plus-end as an inactive motor. In comparison, kinesin is excluded from binding to a TRAK adaptor actively transported by dynein-dynactin towards the minus-end (Fig. 4h).

### Miro1 forms a complex with kinesin, dynein, and TRAK

**Fig. 5:**
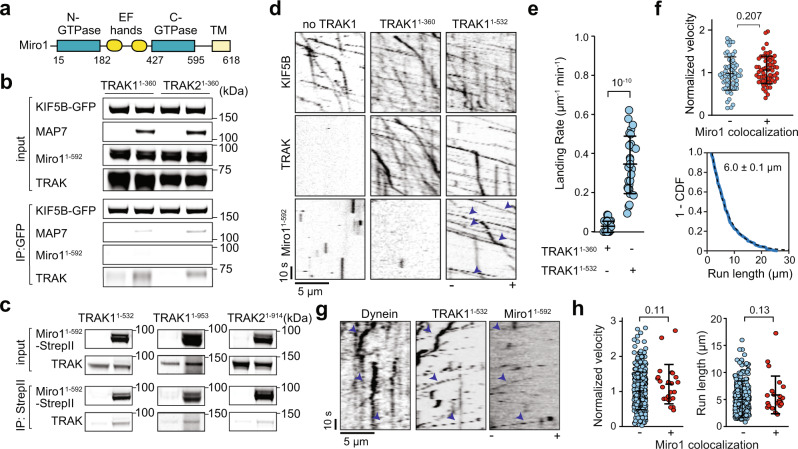
Miro1 is transported by KT and DDT complexes. **a** Miro1 binds to GTP at its GTPase domains and Ca^2+^ ions at its EF-hands and localizes to the outer mitochondrial membrane through its transmembrane (TM) domain. **b** In vitro immunoprecipitation of purified KIF5B-GFP-SNAPf, TRAK1^1–360^ or TRAK2^1–360^, Miro1^1–592^ with or without MAP7. **c**, In vitro immunoprecipitation of purified Miro1^1–592^-StrepII and either TRAK1^1–532^, full-length TRAK1^1–953^, or full-length TRAK2^1–914^ in the presence of GTPγS. KIF5B-GFP-SNAPf was present in all conditions. **d** Kymographs of Alexa488-KIF5B and LD655-Miro1^1–592^ in the absence of a TRAK adaptor or the presence of LD555-TRAK1^1–360^ or TRAK1^1–532^. Assays were conducted at 10 nM MAP7. Arrowheads show colocalization of Miro1 to TRAK. **e** The landing rate of KTM co-localizers in the presence of different TRAK constructs (*n* = 27 MTs for each condition, three independent trials). **f** (Top) Normalized velocity distribution of KT complexes that localize or not localize with Miro1 (*n* = 65 and 61 from left to right). (Bottom) 1-CDF of motor run length for KTM complexes (*n* = 209, three independent experiments). Fits to a single exponential decay (dashed curves) reveal the motor run length (±s.e.). **g** Representative kymographs of LD488-dynein, LD555-TRAK1^1–532^, and LD655-Miro1^1–592^ in the presence of unlabeled dynactin and 1 µM Lis1. The arrowheads show colocalization of dynein, TRAK1, and Miro1. **h** The velocity and run length of DDT_1_^1–532^ that colocalize or not colocalize with Miro1 (*n* = 349 and 23 from left to right). Assays were conducted in 1 µM Lis1 and the absence of Ca^2+^. In (**e**), (**f**), and (**h**), the center line and whiskers represent mean and s.d., respectively. P-values are calculated from a two-tailed t-test.

We next turned our attention to the association of Miro1 with the KT and DDT complexes. Miro1 was shown to interact with TRAK adaptors and kinesin in immunoprecipitation assays^4,5,17^, but it remained unclear which of these interactions form a stable complex capable of plus-end directed transport. To address this question, we expressed a soluble human Miro1 construct lacking its transmembrane domain (Miro1^1–592^, Miro1 hereafter, Fig. 5a, Supplementary Fig. 6a). We first tested whether Miro1 can interact with kinesin independent of TRAK, as previously reported^5^. In pull-down assays using purified components, kinesin pulled down TRAK but not Miro1 when we used TRAK1^1–360^ and TRAK2^1–360^ constructs that bind kinesin but lack the Miro1 interaction domain (Fig. 5b). However, longer (TRAK1^1–532^) or full-length TRAK constructs (TRAK1^1–953^, TRAK2^1–914^) co-precipitated with Miro1 (Fig. 5c). These interactions were stable in the presence of a nonhydrolyzable GTP analog (GTPγS) or GDP (Supplementary Fig. 6b), indicating that Miro1’s nucleotide state does not affect its association with TRAK^39^. Consistent with the pull-down assays, we observed little to no comigration of Miro1 with kinesin motors in the absence of a TRAK adaptor or the presence of 10 nM TRAK1^1–360^ or TRAK2^1–360^ (Fig. 5d, Supplementary Fig. 6c). Unlike TRAK1^1–360^, TRAK1^1–532^ and full-length TRAK1 facilitated the formation of frequent kinesin- TRAK1^1–532^-Miro1 (KTM) complexes that walk along the MTs (Fig. 5d-f, Supplementary Fig. 6d,e). Collectively, these results show that kinesin does not interact with Miro1 in the absence of a TRAK adaptor and that N-terminal 532 residues of TRAK is sufficient to establish a link between kinesin and Miro1.

The velocity and run length of KTM complexes were similar to KT complexes that do not colocalize with Miro1 (Fig. 5f). We also observed dynein-dynactin assembled with TRAK1^1–532^ to comigrate with Miro1 in motility assays (Fig. 5g, Supplementary Fig. 6d). Similar to kinesin, the velocity and run length of DDT_1_^1–532^ complexes that comigrate with Miro1 (DDTM) were similar to DDT_1_^1–532^ only (Fig. 5h). We concluded that Miro1 binding to TRAK1 does not substantially affect the assembly and motility of KT and DDT complexes.

### Ca^2+^-mediated arrest of the mitochondrial transport

We used our in vitro reconstitution assay to test the predictions of the models that explain how Ca^2+^ binding to Miro1 stalls mitochondrial transport. The motor detachment model^5^ predicts that kinesin decouples from Miro1/TRAK in excess Ca^2+^ (Fig. 6a). However, our in vitro immunoprecipitation assays showed that kinesin co-precipitated with TRAK1^1–532^ and Miro1 in the absence or presence of physiological (100 nM) or excess (2 mM) Ca^2+^ (Fig. 6b). Similarly, in the absence of Miro1, kinesin efficiently transports both TRAK1^1–360^ and TRAK2^1–360^ in motility assays, and the percentage of kinesins comigrating with TRAK remain unaffected by the addition of 2 mM Ca^2+^ (Fig. 6c,d). In the presence of Miro1, kinesin and TRAK1^1–532^ comigrated with Miro1 in 0, 0.1, and 2 mM Ca^2+^ (Fig. 6e, Supplementary Fig. 7a, Supplementary Videos 4 and 5). The landing rate, velocity, and run length of KTM complexes in 2 mM Ca^2+^ (Fig. 6f-g) were similar to the no Ca^2+^ condition (Fig. 5e,f). These results are inconsistent with the motor detachment model and show that Miro1 remains bound to the KT complex in the presence of Ca^2+^.

**Figure:**
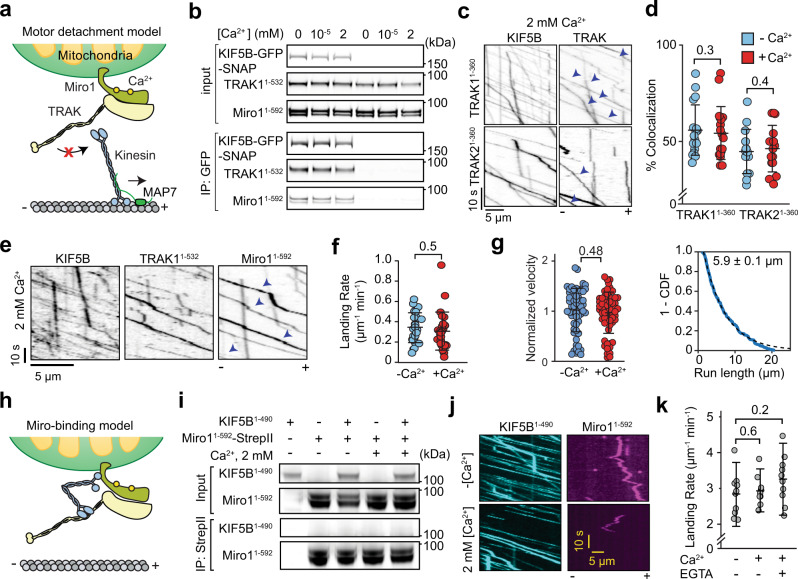
Fig. 6 Kinesin-driven transport of Miro1/TRAK1 is not disrupted by Ca^2+^. **a** The motor detachment model predicts that Ca^2+^ binding to Miro1 triggers dissociation of kinesin from TRAK. **b** In vitro immunoprecipitation of KIF5B-GFP-SNAPf, TRAK1^1–532^, and Miro1^1–592^ in 0, 100 nM, or 2 mM Ca^2+^. **c** Representative kymographs show colocalization of LD555-KIF5B and LD655-TRAK adaptors in 2 mM Ca^2+^ and 10 nM MAP7. Arrowheads show colocalization of TRAK to processive kinesins. **d** The percentage of kinesin runs that colocalize with TRAK on MTs in the presence or absence of 2 mM Ca^2+^ (*n* = 15, 15, 12, and 12 MTs from left to right, three independent trials). **e** A representative kymograph of Alexa488-KIF5B, LD555-TRAK1^1–532^, and LD655-Miro1^1–592^ in 2 mM Ca^2+^ and 10 nM MAP7. Arrowheads show colocalization of Miro1 to TRAK. **f**, The landing rate of KTM complexes in the presence or absence of 2 mM Ca^2+^ (*n* = 27 MTs for each condition, three independent trials). **g** (Left) Normalized velocity distribution of KTM complexes in the presence or absence of 2 mM Ca^2+^. (Right) 1-CDF of motor run length for KTM complexes in 2 mM Ca^2+^ (*n* = 189, three independent experiments). Fits to a single exponential decay (dashed curves) reveal the motor run length (±s.e.). **h** The Miro-binding model predicts that Ca^2+^ binding to Miro1 triggers the binding of the kinesin motor domain to Miro1. **i** In vitro immunoprecipitation assays show no interaction between purified KIF5B^1–490^ and Miro1^1–592^-SNAPf-StrepII in the presence and absence of 2 mM Ca^2+^. **j** Kymographs show that KIF5B^1–490^ does not colocalize with Miro1 in the presence or absence of 2 mM Ca^2+^. Assays were performed in the absence of TRAK adaptors and MAP7. **k** The landing rate of KIF5B^1–490^ in the presence or absence of 2 mM Ca^2+^ and Ca^2+^-chelating agent EGTA (*n* = 10 MTs for all conditions, three independent trials). In (**d**), (**f**), (**g**), and (**k**), the center line and whiskers represent mean and s.d., respectively. *P* values are calculated from a two-tailed t-test.

The Miro-binding model predicts that Ca^2+^ binding causes Miro1 to directly interact with the kinesin motor domain and inhibit kinesin motility^17^ (Fig. 6h). We tested whether Miro1 directly binds and inhibits the motility of a kinesin construct that contains the motor domain but lacks the tail domain (KIF5B^1–490^, Supplementary Fig. 7b). Because KIF5B^1–490^ is a constitutively active motor^32^, these assays were performed in the absence of MAP7. Unlike full-length kinesin, Miro1 did not co-precipitate with KIF5B^1–490^ in the presence or absence of Ca^2+^ (Fig. 6i). In motility assays, KIF5B^1–490^ did not comigrate with Miro1 in the presence or absence of Ca^2+^ (Fig. 6j and Supplementary Fig. 7c). In addition, the landing rate of KIF5B^1–490^ on MTs remained unaffected by the addition of Miro1 and 2 mM Ca^2+^ (Fig. 6k). Collectively, our results are inconsistent with the Miro-binding model and show that Miro1 does not bind and inhibit the kinesin motor domain in response to elevated Ca^2+^.

**Fig. 7:**
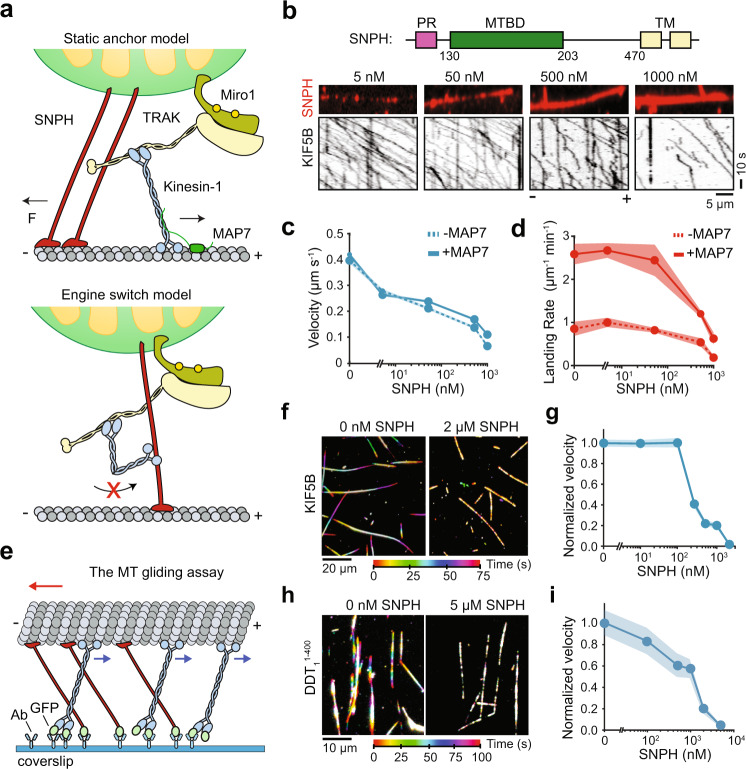
SNPH stalls MT gliding driven by kinesin and dynein motors. **a** Static anchor, and engine switch models for SNPH-mediated stalling of mitochondrial transport. **b** (Top) Domain organization of human SNPH (MTBD: MT-binding domain, PR: proline-rich domain). (Middle) SNPH decorates surface-immobilized MTs. (Bottom) Representative kymographs of kinesin motility in the presence of increasing concentrations of SNPH. The assays were conducted in 10 nM MAP7. **c** The average velocity of kinesin on SNPH-decorated MTs in the presence (± s.e.m., *n* = 98, 158, 95, 58, and 20 from left to right) and absence of 10 nM MAP7 (*n* = 115, 191, 154, 93, and 59 from left to right). **d** The landing rate of kinesin onto SNPH-decorated MTs in the presence and absence of 10 nM MAP7 (mean ± s.e.m., *n* = 9 MTs for each condition, three independent trials). **e** Schematic of the MT gliding by kinesin motors. Motors were fixed on the glass surface from their tail through a GFP-antibody linkage. MTs glide with their minus-ends in the lead (red arrow) due to the plus-end directed motility of kinesins (blue arrows). Static binding of SNPH to MT exerts resistive forces against gliding motility. **f** Representative color-coded time projections of Cy5-MTs glided by KIF5B-GFP-SNAPf in the presence or absence of 2 µM SNPH-sfGFP. **g** MT gliding velocities of kinesin motors under increasing SNPH-sfGFP concentration (mean ± s.e.m., *n* = 50, 57, 58, 59, 71, 70, 61 MTs from left to right, two independent trials). **h**, Representative color-coded time projections of Cy5-MTs with DDT assembled with TRAK1^1–400^-GFP in the presence or absence of 5 µM SNPH-sfGFP. Assays were conducted in 1 µM Lis1. **i** MT gliding velocities of DDT complexes under increasing SNPH-sfGFP concentration (mean ± s.e.m., *n* = 60, 61, 67, 72, 64, 60 MTs from left to right, three independent trials).

Finally, we tested the models that propose Ca^2+^-mediated accumulation of SNPH to the outer mitochondrial membrane to inhibit both anterograde and retrograde transport of mitochondria^18,20^. The “engine-switch and brake” model predicts that SNPH inhibits kinesin through direct molecular interaction^20^ (Fig. 7a). To test this model, we expressed an SNPH construct that lacks the C-terminal transmembrane domain (SNPH^1–473^, Supplementary Fig. 8a). We did not observe strong interactions between kinesin and SNPH in immunoprecipitation assays (Supplementary Fig. 8b). In motility assays, we confirmed that SNPH densely decorates the MT surface^18^ (Fig. 7b). According to the “engine-switch and brake” model, decoration of the MT surface by SNPH would increase the kinesin landing rate but prevent subsequent motility. However, kinesin was able to walk along the MT even in the presence of 1 µM SNPH, indicating that SNPH does not inhibit kinesin motility through direct interactions. We note that the addition of SNPH substantially decreased the kinesin landing rate to MTs and slowed down subsequent motility (Fig. 7b-d and Supplementary Video 6), raising the possibility that MT binding of SNPH may reduce MT recruitment and velocity of kinesin similar to MT-associated proteins (MAPs)^40^.

The “static anchor” model predicts that SNPH can induce resistive forces against motility by passively anchoring mitochondria to MTs (Fig. 7a)^18,20^. To test this prediction, we immobilized kinesin and SNPH to the glass surface and asked how MT binding of SNPH affects MT gliding activity of kinesin and dynein motors (Fig. 7e). Consistent with this model, the addition of SNPH slowed down gliding motility driven by kinesin (Fig. 7f-g and Supplementary Video 7) and DDT_1_^1–400^ in a dose-dependent manner (Fig. 7h,i). MT gliding was almost completely stopped when we incubated the glass surface with excess (1-2 µM) SNPH (Fig. 7g and Supplementary Fig. 8c,d), suggesting that multiple SNPH molecules may be required to efficiently counter either motor. These results support the model that SNPH inhibits both anterograde and retrograde transport of mitochondria by passively anchoring mitochondria to MTs^18,20^.

## Discussion

**Fig. 8:**
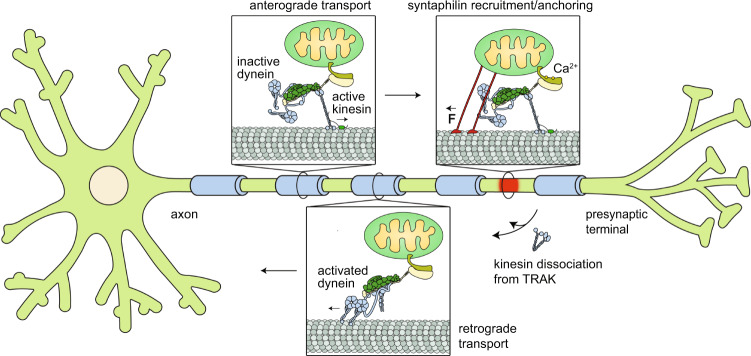
A model for bidirectional transport and Ca^2+^-mediated arrest of mitochondria in neurons. Mitochondria are transported anterogradely by active kinesin motors recruited by TRAK1 while dynein-dynactin is transported as an inactive passenger. In regions with high Ca^2+^ concentration (red), mitochondria recruit SNPH, which anchors the mitochondria to the MT and stalls the transport machinery. Retrograde transport is initiated by the dissociation of kinesin from TRAK, followed by activation of the DDT complex.

In this study, we used in vitro reconstitution to demonstrate that kinesin and dynein transport Miro1/TRAK1 towards the plus- and minus-ends of MTs. Our work provides an explanation for how opposing actions of kinesin and dynein might be regulated by TRAK adaptors to control the directionality of mitochondrial transport (Fig. 8). We showed that TRAK1 and TRAK2 are bona fide adaptors that activate the motility and force generation of dynein-dynactin. Consistent with studies in neurons^7^, TRAK1, and to a lesser extent TRAK2, recruit kinesin, but TRAK binding is not sufficient to substantially activate kinesin-1 for processive motility. We observed that MAP7 decoration of MTs promoted the motility of KT complexes, highlighting the necessity of MAP7 for most, if not all, kinesin-1-driven transport in various cell types^32,41^. In the presence of MAP7, kinesin binding to TRAK1 increased its MT landing rate and run length. In addition, an increase in TRAK concentration stimulated more frequent kinesin motility at a given MAP7 concentration, indicating that TRAK binding may increase the stability of the active conformation of kinesin once this motor is activated by MAP7. These results are in agreement with a recent in vitro reconstitution study^42^, which showed that kinesin-1 employs a two-step activation process that involves binding to a cargo adaptor and interacting with MAP7 on MTs.

Our results are largely consistent with recent reports that studied kinesin and dynein-mediated transport of TRAK1 and TRAK2 adaptors in vitro. Similar to our results, Henrichs et al. showed that full-length TRAK1 activates kinesin motility and increases the motor run length^14^. This study has reported activation of kinesin by full-length TRAK1 without MAP7^14^. While we also observed occasional processive runs of kinesin by TRAK1 addition in the absence of MAP7, the landing rate of these runs was ~40-fold lower compared to the addition of both TRAK1 and MAP7. Henrichs et al. also attributed the increase in the processivity of KT complexes to the interaction of the TRAK1 C-terminus with MTs^14^. However, we observed that TRAK1 only weakly interacts with MTs at high concentrations, consistent with the lack of strong MT localization of TRAK adaptors in cells^7^. We also observed that TRAK binding increases kinesin run length even in the absence of the TRAK1 C-terminus, suggesting that enhanced processivity of KT complexes is not directly related to MT binding of TRAK1.

More recently, Fenton et al. reported TRAK2-mediated activation of kinesin and dynein-dynactin motility in cell extracts and observed that TRAK2 adaptors that recruit both kinesin and dynein exclusively moved towards the MT plus-end^15^. We also observed our reconstituted DDKT complexes to exclusively move towards the plus-end at speeds comparable to kinesin, indicating that dynein is transported as an inactive passenger by kinesin on TRAK adaptors (Fig. 8). This is consistent with the observation that mitochondria move at similar speeds to kinesin in an anterograde direction^43^ even though dynein is localized to these cargos^44,45^. Fenton et al. reported that the knockdown of either kinesin or dynein also results in a decrease in the frequency of TRAK2 transport in the opposite direction^15^, indicating that TRAK binding of one motor increases the affinity of the other motor to the same TRAK adaptor. Our results are not fully consistent with this view, because we did not observe dynein transporting kinesin towards the minus-end. Instead, the assembly of the active DDT complexes was mutually exclusive with kinesin binding to the TRAK adaptor. While we cannot exclude the possibility that our purified system lacks additional factors that link these motors together on TRAK, these observations suggest that retrograde transport of Miro1/TRAK requires dissociation of kinesin and formation of active dynein-dynactin complex on TRAK (Fig. 8). Our results do not exclude the possibility that kinesin remains bound to retrogradely moving mitochondria by interacting with other mitochondrial adaptor proteins and these possibilities remain to be tested by tracking the association of motor proteins with anterogradely and retrogradely transported mitochondria in live cells^46^.

The motility of DDKT complexes was markedly different from the reconstituted kinesin-3/Hook3/dynein-dynactin complex^47^. Unlike DDKT, complexes assembled with Hook3 move either towards the plus- or minus-end of the MT at speeds lower than kinesin or dynein alone^47^, suggesting that the presence of the opposing motor slows down the motility of the active motor. These differences may be attributed to whether kinesin and dynein motors interact with each other when they are bound to the same adaptor. While kinesin-3 and dynein bind to distant sites on Hook3^47^, kinesin-1 and dynein may have overlapping binding sites on TRAK. Although the kinesin binding site on TRAK1 has not been well characterized, the coiled-coil between the CC1 box and the Spindly motif (amino acids 100-360) of TRAK1 was sufficient to co-precipitate with kinesin^7^. Based on the structures of dynein-dynactin assembled with BicD or Hook family adaptors^36,48^, this entire region is expected to run from the pointed to the barbed end of dynactin in active DDT complexes. If kinesin has a smaller footprint on TRAK, it may block some of the pairwise interactions required for the formation of the active DDT complex, but not fully inhibit the binding of dynein and dynactin to the rest of this coiled-coil. In comparison, activation of the DDT complex may block the entire kinesin binding site, and thereby, exclude kinesin from motile DDT complexes. Alternatively, TRAK may coordinate motor binding through a registry shift of its coiled coils, as proposed for the BicD2 adaptor^49^. These possibilities could be best distinguished by future cryo-electron imaging of reconstituted DDKT complexes at near-atomic resolution.

The in vitro reconstitution assay we developed enabled us to directly test the predictions of the models that describe how elevated levels of intracellular Ca^2+^ arrest mitochondrial transport. We first tested whether Miro1 dissociates from or inhibits the KT complexes in the presence of excess Ca^2+^. The assembly and motility of the KTM complexes were unaffected by increased Ca^2+^ levels, which is incompatible with both ‘motor detachment’ and ‘Miro binding’ models^5,17^. Our results indicate that Ca^2+^ binding to Miro1 does not directly disrupt the machinery that transports mitochondria, and thereby Ca^2+^-mediated docking of mitochondria might occur upstream of the motor transport machinery (Fig. 8). However, we provided evidence that the MT anchoring protein SNPH is sufficient to stall the MT gliding activity of kinesin and dynein motors, consistent with overexpression of this protein inhibiting back and forth transport of mitochondria in axons^18^.

We note that our results do not discount the role of Miro1 to coordinate the arrest of mitochondrial transport. Miro1 may serve as the primary factor that facilitates Ca^2+^-mediated recruitment of SNPH^18^ and other associated factors to the outer mitochondrial membrane. In particular, the dynein-interacting protein Disrupted in Schizophrenia 1 (DISC1)^50^, Armadillo repeat-containing X-linked (Armcx) 1 and 3^51,52^, and mitochondrial fusion proteins MFN1 and MFN2 have been shown to interact with the Miro1/TRAK complex, and knockdown of these factors led to defects in mitochondrial transport^46^. The in vitro reconstitution assay we developed provides an experimental platform to investigate the molecular mechanism of how these factors regulate mitochondrial transport.

## Methods

### Cloning and plasmid generation

The constructs expressing the phi-dynein mutant (SNAP–DHC R1567E-K1610E), full-length TRAK1, and full-length TRAK2 were provided in a pACEBac1 vector backbone by A.P. Carter (MRC, University of Cambridge). The sequences encoding full-length or truncated versions of human TRAK1 and TRAK2 were cloned into the pOmniBac vector. All constructs contained an N-terminal His6-ZZ tag followed by a TEV protease cleavage site for protein purification and a C-terminal SNAP-tag or GFP fusion for labeling and imaging purposes. A cDNA for full-length human kinesin (*KIF5B*; amino acids 1–963, clone ID 8991995) was obtained from GE Dharmacon and fused to GFP-SNAPf at its C terminus. The phi mutant of the dynein-1 heavy chain (DHC) was co-expressed by fusing the coding sequence to the pDyn2 plasmid containing genes encoding IC2C, LIC2, TCTEX1, LC8, and ROBL1, as described^26^. The list of constructs used for each dataset is given in Supplementary Table 1.

### Protein expression and purification

Native dynactin was purified from pig brains (Yosemite Foods)^53^. Fresh brains were purchased from a butcher and transported in ice-cold PBS. Brains were washed in homogenization buffer (35 mM PIPES pH 7.2, 5 mM MgSO_4_, 1 mM EGTA, 0.5 mM EDTA) and frozen in liquid nitrogen. Three frozen brains were broken into pieces and blended in a blender in the presence of homogenization buffer supplemented with four EDTA protease-inhibitor tablets per 500 mL (Roche), 1.6 mM PMSF, 1 mM DTT, and 0.1 mM ATP. The brain lysate was gently stirred for 30 min at 4°C until completely thawed and was then spun in a JLA 8.1 fixed angle rotor (Beckman Coulter) at 8,000 r.p.m. for 45 min at 20 °C. The supernatant was collected and further clarified using a Type 45 T.i. rotor (Beckman Coulter) at 45,000 r.p.m. for 50 min at 4°C. The supernatant was then filtered with a glass-fiber filter (Sartorius), followed by a 0.45 μm filter (Milex Millipore). The sample was loaded into a SP-Sepharose column (Cytiva) pre-equilibrated with SP buffer (35 mM PIPES pH 7.2, 5 mM MgSO_4_, 1 mM EGTA, 0.5 mM EDTA, 1 mM DTT, and 0.1 mM ATP) using an Akta FPLC (Cytiva). The column was washed with 4 column volumes of SP buffer in the presence of 3 mM KCl followed by elution using a linear gradient up to 250 mM KCl. Fractions collected from the first major peak were pooled, filtered using a 0.22 μm filter (Milex Millipore), then loaded onto a MonoQ 16/10 column (Cytiva) pre-equilibrated with MonoQ buffer (35 mM PIPES pH 7.2, 5 mM MgSO_4_, 1 mM EGTA, 0.5 mM EDTA, 1 mM DTT, and 0.1 mM ATP). The column was washed with 10 column volumes of MonoQ buffer followed by elution using a linear gradient up to 150 mM KC1 in 1 column volume, a second linear gradient up to 350 mM KCl in 10 column volumes, and a third linear gradient up to 1 M KCl in 1 column volume. The peak dynactin fractions located at approximately 38 mS cm^−1^ were then pooled and concentrated to a volume of about 3 mL and loaded onto a G4000_SW_ 21.5/600 column (Tosoh Bioscience) equilibrated with GF150 buffer (25 mM HEPES pH 7.2, 150 mM KCl, 1 mM MgCl_2_, 5 mM DTT, and 0.1 mM ATP) for gel-filtration. The dynactin peak collected, pooled, and concentrated to approximately 2 mg mL^−1^ using an 0.5 mL 100 kDa MWCO filter unit (Millipore). 2 μL aliquots were then flash frozen in liquid nitrogen and stored at −80 °C.

The phi mutant of dynein, KIF5B, MAP7, and truncated TRAK1, and TRAK2 constructs were purified from baculovirus-infected SF9 cells (UC Berkeley Cell Culture Facility). Briefly, cell pellets were resuspended in a lysis buffer (see below) supplemented with cOmplete Protease Inhibitor Cocktail (Roche) and lysed using a dounce homogenizer (Wheaton) (20 strokes with loose plunger followed by 20 strokes tight plunger). The cell lysate was clarified by centrifugation at 186,000 g for 45 min, incubated with IgG Sepharose (Cytiva) for 2 hr at 4 °C, applied to a gravity flow column, and washed extensively with a TEV wash buffer (see below). The protein-bead complexes were then treated with TEV protease (UC Berkeley MacroLab, Addgene Cat#8827) at 12 °C overnight. The mixture was then centrifuged at 4000 g for 5 min and the supernatant was concentrated using an Amicon Ultra 0.5 mL spin column (EMD Millipore). Protein concentration was determined by measuring the OD_280_ using Nanodrop 1000 (Thermofisher).

Different buffer conditions were used for each protein preparation. Cells expressing dynein were lysed in a dynein lysis buffer (50 mM HEPES pH 7.4, 100 mM NaCl, 10% Glycerol, 1 mM DTT, 2 mM PMSF), IgG beads (Cytiva) were washed with a dynein TEV wash buffer (50 mM Tris-HCl pH 7.4, 150 mM K-Acetate, 2 mM Mg-Acetate, 1 mM EGTA, 10% Glycerol, 1 mM DTT) and the protein was concentrated using a 100 K molecular weight cut-off (MWCO) spin filter (EMD Millipore). For kinesin purification, the cells were lysed in a kinesin lysis buffer **(**50 mM HEPES pH 7.4, 1 M NaCl, 10% Glycerol, 1 mM DTT, 2 mM PMSF), IgG beads were washed with a kinesin TEV wash buffer (50 mM HEPES pH 7.4, 300 mM NaCl, 10% Glycerol, 1 mM EGTA, 1 mM DTT), and the protein was concentrated using 100 K MWCO spin filter. MAP7 purification was performed using a MAP7 lysis buffer (25 mM HEPES pH 7.4, 1 M KCl, 10% Glycerol, 1 mM DTT, 1 mM PMSF) and MAP7 TEV wash buffer (25 mM HEPES pH 7.4, 300 mM KCl, 1 mM EGTA, 10 mM MgCl_2_, 10% Glycerol, 1 mM DTT). MAP7 was concentrated using a 50 K MWCO spin filter. Truncated TRAK constructs were purified in the presence of high salt, glutamic acid, and arginine to improve solubility (TRAK lysis buffer: 50 mM HEPES pH 7.4, 1 M NaCl, 10% Glycerol, 50 mM L-Glu, 50 mM L-Arg, 1 mM DTT, 2 mM PMSF, 1 mM ATP; and TRAK TEV wash buffer: 50 mM HEPES pH 7.4, 500 mM NaCl, 50 mM L-Glu, 50 mM L-Arg, 10% Glycerol, 1 mM EGTA, 1 mM DTT, 1 mM ATP), and protein was concentrated using a 50 K MWCO spin column. Full-length TRAK1 and TRAK2 were purified from HEK 293 F GNTI^-/-^ cells (UC Berkeley Cell Culture Facility) using polyethyleneimine (PEI, Sigma-Aldrich) transfection of the pcDNA and the TRAK purification procedure described above.

Miro1^1–592^ (Miro1^1–592^-SNAP-psc-StrepII) was purified using Miro lysis buffer (25 mM HEPES pH 7.4, 300 mM NaCl, 10% Glycerol, 1 mM DTT, 1 mM PMSF) supplemented with 1x protease inhibitor cocktail (Roche) and lysed using a dounce homogenizer. The lysate was clarified by centrifugation at 65,000 g for 45 min, incubated with Streptactin Sepharose beads (IBA) for 2 hr at 4 °C, applied to a gravity flow column, and washed extensively with Miro wash buffer (25 mM HEPES pH 7.4, 300 mM NaCl, 10% Glycerol, 1 mM DTT). The protein was then eluted from beads with 3 mM desthiobiotin and concentrated using a 50 K MWCO spin column (EMD Millipore).

SNPH^1–473^ was purified from BL21(DE3) *E. coli* cells (UC Berkeley QB3 MacroLab). Briefly, cell pellets were resuspended in SNPH lysis buffer (25 mM HEPES pH 7.4, 300 mM NaCl, 10% Glycerol, 1 mM DTT, 1 mM PMSF) supplemented with 1x protease inhibitor cocktail (Roche) and lysed using a tip sonicator (Branson) for 2 min. The lysate was clarified by centrifugation at 65,000 g for 45 min, incubated with IgG Sepharose beads for 2 h at 4 °C, applied to a gravity flow column, and washed extensively with SNPH wash buffer (25 mM HEPES pH 7.4, 300 mM NaCl, 10% Glycerol, 1 mM DTT). The protein-bead complexes were then treated with TEV protease at 4 ^o^C overnight. The mixture was then centrifuged at 4000 g for 5 min and the supernatant was concentrated using a 50 K MWCO spin column (EMD Millipore).

### Co-immunoprecipitation assays

For each sample, 10 µg of each protein construct was pre-mixed on ice for 5 min and then added to 15 µL of GFP-Trap beads (Chromotek) that were pre-washed with the MB buffer (30 mM HEPES, 5 mM MgSO_4_, 1 mM EGTA, pH 7.0). Samples were then diluted 1:2 in MB supplemented with 100 mM NaCl, 1 mg ml^−1^ BSA, and 1 mM DDT and incubated on ice for 2 h to facilitate complex formation. 10% of samples were collected for input lanes and then washed 3x with the MB buffer including supplements. Samples were then resuspended into LDS sample buffer (Invitrogen), boiled for 10 min at 95 ^o^C, and run on an SDS-PAGE gel. Imaging was performed on a GE Typhoon FLA fluorescence imager.

### Labeling

Proteins were labeled with fluorescent probes before they were eluted from the affinity columns. For SNAP labeling, IgG bead slurry (Cytiva) was concentrated to 5 mL, followed by the addition of 5 nmol of either BG-LD555 or BG-LD655 dye (Lumidyne), followed by incubation for 1 h at 4 ^o^C. The slurry was added to a gravity flow column and washed extensively in a wash buffer. For ybbR labeling, bead slurry was concentrated to 5 mL, followed by the addition of 5 nmol of either CoA-LD555 or BG-LD655 dye (Lumidyne) followed by incubation for 30 min at room temperature in the presence of 1 μM Sfp phosphopantetheinyl transferase (Addgene #75015)to catalyze protein labeling.

### Motility assays

To immobilize biotinylated MTs to the coverslip, 1 mg ml^−1^ BSA-biotin (Sigma) was introduced into the flow chamber, which was then washed with MB buffer supplemented with 1 mM DTT, 10 μM taxol, 1.25 mg ml^−1^ casein (Sigma) and 0.5% pluronic (MBCT). MBCT was additionally supplemented with 0.2% methylcellulose and 50 nM K-Acetate for DDKT motility assays and dynein motility assays with TRAK1^1–532^. The chamber was then incubated with 20 µl 1 mg ml^−1^ streptavidin (NEB) in MBCT and washed with 40 µl MBCT. For imaging dynein motility, fluorescently-labeled dynein, dynactin, and a cargo adaptor (TRAK1 or TRAK2) were mixed at a 1:5:20 molar ratio in MB for TRAK1^1–400^ and TRAK2^1–400^ and a 1:3:10 molar ratio for TRAK1^1–532^, respectively. Miro1^1–592^, dynein, dynactin, and TRAK1^1–532^ were mixed at a 1:3:10:30 molar ratio, respectively, in the presence of 1 μM Lis1. For imaging kinesin motility, kinesin, a cargo adaptor (TRAK1 or TRAK2), MAP7, and Miro1^1–592^ were mixed at a 1:3:3:3 molar ratio, respectively, in MB buffer. For imaging both dynein and kinesin simultaneously, dynein, dynactin, kinesin, and a cargo adaptor were mixed at a 1:3:1:1 (TRAK1) or 1:5:1:1 (TRAK2) ratios, respectively, in MB buffer with the stated concentration of MAP7. The mixtures were incubated on ice for 10 min and diluted 30-fold in MBCT. Finally, the mixture was diluted 10-fold in the stepping buffer (MBCT supplemented with 0.1 mg ml^−1^ glucose oxidase (Sigma), 0.02 mg ml^−1^ catalase (Sigma), 0.8% D-glucose, and 1 mM Mg·ATP) and introduced into the chamber. Motility was recorded for 5 min. For assays including Miro1^1–592^, 0.1 mg ml^−1^ biotin-BSA was also included in the stepping buffer for surface passivation.

### Gliding assays

Rabbit monoclonal anti-GFP antibody (~0.4 mg ml^−1^, Covance) was flown into an assay chamber and incubated for 3 min. The chamber was washed with 30 μl MB supplemented with 1 mM DTT, 10 μM taxol, and 1.25 mg ml^−1^ casein. For kinesin-driven MT gliding, 10 μl of 2.5 nM GFP-tagged kinesin was subsequently added to the chamber. In the case of DDT-driven MT gliding, 10 nM dynein, 10 nm dynactin, and 10 nM GFP-TRAK2^1–400^ were incubated in MB on ice for 10 min in the presence of 1 µM Lis1, and 10 μl of this mixture were added to the chamber. After 2 min incubation, the unbound motor was removed by washing the chamber with 30 μl MB. For experiments with SNPH, 10 μl of SNPH^1–473^-sfGFP was added to the chamber at the indicated concentration for 2 min followed by a 30 μl MB wash. Then, 10 μl of 200 nM Cy5-labeled MTs were flown to the chamber and allowed to bind the kinesin- or DDT-decorated surface for 4 min. The chamber was then washed with 60 μl MB. Lastly, 10 μl of imaging buffer (MB supplemented with 0.02 mg ml^−1^ catalase, 0.8% D-glucose, and 1 mM Mg·ATP) was flown into the chamber to initiate gliding motility.

### Microscopy

Fluorescence imaging experiments were performed using a custom-built multicolor TIRF setup equipped with a Ti-Eclipse inverted microscope body, a 100X magnification 1.49 N.A. apochromat oil-immersion objective (Nikon), a perfect focusing system, and an electron-multiplied charge-coupled device camera (Andor, Ixon EM+, 512 × 512 pixels) with an effective pixel size of 160 nm after magnification. Alexa488/GFP, LD555, and LD655 probes were excited with fiber-coupled 0.05 kW cm^−2^ 488-nm, 561-nm, and 633-nm laser beams (Coherent) through a Nikon TIRF Illuminator, and their fluorescent emissions were filtered through a notch dichroic filter and 525/40, 585/40, and 655/40 bandpass emission filters (Semrock), respectively. Multicolor fluorescence imaging was performed using the time-sharing mode in MicroManager. Videos were recorded at 2-4 Hz.

### Data Analysis

Coiled-coil prediction scores of human TRAK1 and TRAK2 were calculated from the NPS@ server using the algorithm of Lupas et al.^54^. Videos were analyzed in ImageJ. Kymographs were generated by plotting segmented lines along the MTs using a custom-written ImageJ macro. The processive movement was defined and analyzed as described previously^27^. Complexes that exhibited diffusive movement, ran for less than 250 nm, and paused for more than 1 s were excluded from velocity analysis. For two-color imaging, the fluorescence channels were overlaid in ImageJ to generate a composite image. Colocalization events were manually scored in kymographs. For two-color imaging of a motor and a cargo adaptor (TRAK1 or TRAK2), processive motility events observed in the cargo adaptor channel that did not colocalize with a motor were still included in the velocity analysis.

### Optical trapping assays

DDT_1_^1–400^ and DDT_2_^1–400^ complexes were assembled with 1 µl of 0.84 mg ml^−1^ dynein, 1 µl of 1.7 mg ml^−1^ dynactin, and 1 µL of 0.22 mg ml^−1^ TRAK1^1–400^ or 0.1 mg ml^−1^ TRAK2^1–400^ in MB for 5 min at 4°C. The protein mixture was then added to 700 nm diameter polystyrene beads (Invitrogen) coated with a polyclonal GFP antibody (Covance) and incubated for 10 min. Similarly, KT_1_^1–400^ complexes were assembled with 1 µl of 0.05 mg ml^−1^ biotinylated kinesin-ybbR and 1 µl of 0.1 mg ml^−1^ TRAK1^1–400^ before being added to 700 nm diameter streptavidin-coated beads (Spherotech). Flow chambers were first decorated with Cy5-labeled sea urchin axonemes in MB. The motor-bead mixture was introduced to the chamber in the imaging buffer. To ensure that more than ~95% of beads were driven by single motors, the protein mixture was diluted before incubating with beads such that a maximum of 30% of beads exhibited activity when brought into contact with an axoneme.

Optical trapping experiments were performed on a custom-built optical trap microscope set-up controlled using Labview 2017 software^55^. Briefly, motor-coated beads were trapped with a 2 W 1,064-nm laser beam (Coherent) focused on the image plane using a 100X magnification 1.49 N.A. apochromat oil-immersion objective (Nikon). Cy5-labeled sea urchin axonemes were excited with a 633-nm HeNe laser (JDSU Uniphase), imaged using a monochrome camera (The Imaging Source), and moved to the center of the field of view using a locking XY stage (M-687, Physik Instrumente). The trapped bead was lowered to the surface of the axonemes using a piezo flexure objective scanner (P-721 PIFOC, Physik Instrumente). Bead position relative to the center of the trap was monitored by imaging the back-focal plane of a 1.4 N.A. oil-immersion condenser (Nikon) on a position-sensitive detector (First Sensor). Beam steering was controlled with a pair of perpendicular acousto-optical deflectors (AA Opto-Electronic). For calibrating the detector response, a trapped bead was rapidly raster-scanned by the acousto-optical deflector and trap stiffness was derived from the Lorentzian fit to the power spectrum of the trapped bead. The spring constant was set to ~0.04 pN nm^−1^ for DDT_1_^1–400^ and DDT_2_^1–400^, and ~0.08 pN nm^−1^ for KT_1_^1–400^ experiments.

Custom MATLAB software was used to extract stall forces and stall times from raw traces. First, raw traces were downsampled from 5,000 Hz to 250 Hz. Stall events were defined as a stationary period of a motor at forces above 2.5 pN lasting a minimum of 100 ms, followed by snapping back of the bead to the trap center. The stall force was defined as the mean force in the last 20% of the stall event. The stall time was defined as the interval the bead spent at a force of at least 80% of the stall force. All stall events were plotted and manually reviewed to confirm the accuracy of the reported values.

### Statistics and reproducibility

At least two independent repetitions were performed to obtain any given result. The number of replicates (*n*) and statistical analysis methods are clearly stated in the figure legends. Representative data are shown from independently repeated experiments.

### Reporting summary

Further information on research design is available in the Nature Portfolio Reporting Summary linked to this article.

## Supplementary information

Supplementary Information

Description of Additional Supplementary Files

Supplementary Movie 1

Supplementary Movie 2

Supplementary Movie 3

Supplementary Movie 4

Supplementary Movie 5

Supplementary Movie 6

Supplementary Movie 7

Reporting Summary

## Data Availability

A reporting summary for this article is available as Supplementary Information file. The main data supporting the findings of this study are available within the article and its Supplementary Figures. The source data underlying Figs. 1–7, Supplementary Figs. 1, 2, 4-6 and 8 are provided as a Source Data file. Additional details on datasets and protocols that support the findings of this study will be made available by the corresponding authors upon reasonable request. Source data are provided with this paper.

## Source data

Source Data
