# Supplementary material for: TRAK adaptors regulate the recruitment and activation of dynein and kinesin in mitochondrial transport
Source: Nat Commun. 2023 Mar 13;14:1376. doi: 10.1038/s41467-023-36945-8 (PMC10011603; doi:10.1038/s41467-023-36945-8)
Supplement: Supplementary file 1 — Supplementary Information [file 41467_2023_36945_MOESM1_ESM.pdf]

Supplementary Information

Supplementary Tables

**Construct Name**	**Figures**
SNAPf-DYNH1C1$_{R1567E\text{-}K1610E}$ -IC2C-LIC2-Robl1-Tctex1-LC8	Fig. 1c-f; Fig. 3; Fig. 4a-g; Fig. 5g-h; Fig. 7h-i; S1b-c; S4b-d; S5a-d; S6f
TRAK1$^{1\text{-}360}$-SNAPf	Fig. 1c,f; Fig. 2a-f; Fig. 5b,d,e; Fig. 6c,d; S1a,b; S2b; S3a
TRAK1$^{1\text{-}400}$-SNAPf	Fig. 1c,e,f; Fig. 4a-b, e-g; S1a-c; S2c; S5c-d
TRAK2$^{1\text{-}360}$-SNAPf	Fig. 1d,f; Fig. 2a-f; Fig. 5b; S1a-c, S2b; S6c
TRAK2$^{1\text{-}400}$-SNAPf	Fig. 1d-f; S 1a-c; S 2c; Fig. 4c-d, S5a-d
KIF5B-GFP-SNAPf	Fig. 2a-f; Fig. 4a-g; Fig. 5b-f; Fig. 6b-g; Fig. 7b-d,f,g; S2a-e; S5a-d; S6c-e; S7a; S8b
MAP7-ybbR	Fig. 2a-f; Fig. 4a-e; Fig. 5b,d-f; Fig. 6c-g; Fig. 7b-d; S2b-e; S5a-d; S6c-e; S7a; S8b
TRAK1$^{1\text{-}360}$-GFP	S4a,b
TRAK1$^{1\text{-}400}$-GFP	Fig. 3; Fig. 7h-i; S4a-d
TRAK2$^{1\text{-}360}$-GFP	S4a,b
TRAK2$^{1\text{-}400}$-GFP	Fig. 3; S4a-d
TRAK1$^{1\text{-}532}$-SNAPf	Fig. 5c-h; Fig. 6e-g; S3b; S6b,d,h; S7a
KIF5B-ybbR	Fig. 3; S4a,c,d
KIF5B$^{\Delta 1\text{-}336}$-GFP-SNAPf	Fig. 4f,g; S2a
KIF5B$^{1\text{-}490}$-GFP-SNAPf	Fig. 6i-k; S7b,c
Miro1$^{1\text{-}592}$-SNAPf- StrepII	Fig. 5a-h; Fig. 6b,e-g,i-k; S6a-e; S7a,c
TRAK1$^{1\text{-}953}$-SNAPf	Fig. 5c; S2d,e; S3b; S6e
TRAK1$^{1\text{-}953}$-sfGFP-SNAPf	S6b
TRAK2$^{1\text{-}914}$-SNAPf	Fig. 5c; S2d,e; S6b
SNPH$^{1\text{-}473}$-sfGFP	Fig. 7b-d,f-i; S8a,c,d
SNPH$^{1\text{-}473}$-ybbR	S8a,b
Lis1-SNAPf	Fig. 1c,d,f; Fig. 5g,h; Fig. 7h-i; S1b; S5c,d; S6f

Supplementary Table 1 | The list of constructs used in this study. The SNAPf-DYNH1C1$_{R1567E\text{-}K1610E}$ -IC2C-LIC2-Robl1-Tctex1-LC8 construct was a gift from A. Carter (MRC, UK). Other constructs were cloned into the pOmniBac vector for baculovirus expression in SF9 cells, except SNPH constructs which were cloned into the pET17b vector for bacterial expression. A ZZ-Tev tag was inserted at the N-termini of all constructs (except Miro1) for binding the protein to IgG beads and eluting by TEV protease. The SNAPf tag was used to label the proteins with fluorescent dyes or biotin. The ybbR tag was inserted for SFP-catalyzed labeling of proteins with CoA-functionalized fluorescent dyes or biotin. A StrepII tag was inserted at the C-termini of Miro1$^{1\text{-}592}$ to elute the protein with desthiobiotin from Strep-Tactin-coated beads (S: Supplementary Figure).

Supplementary Figures

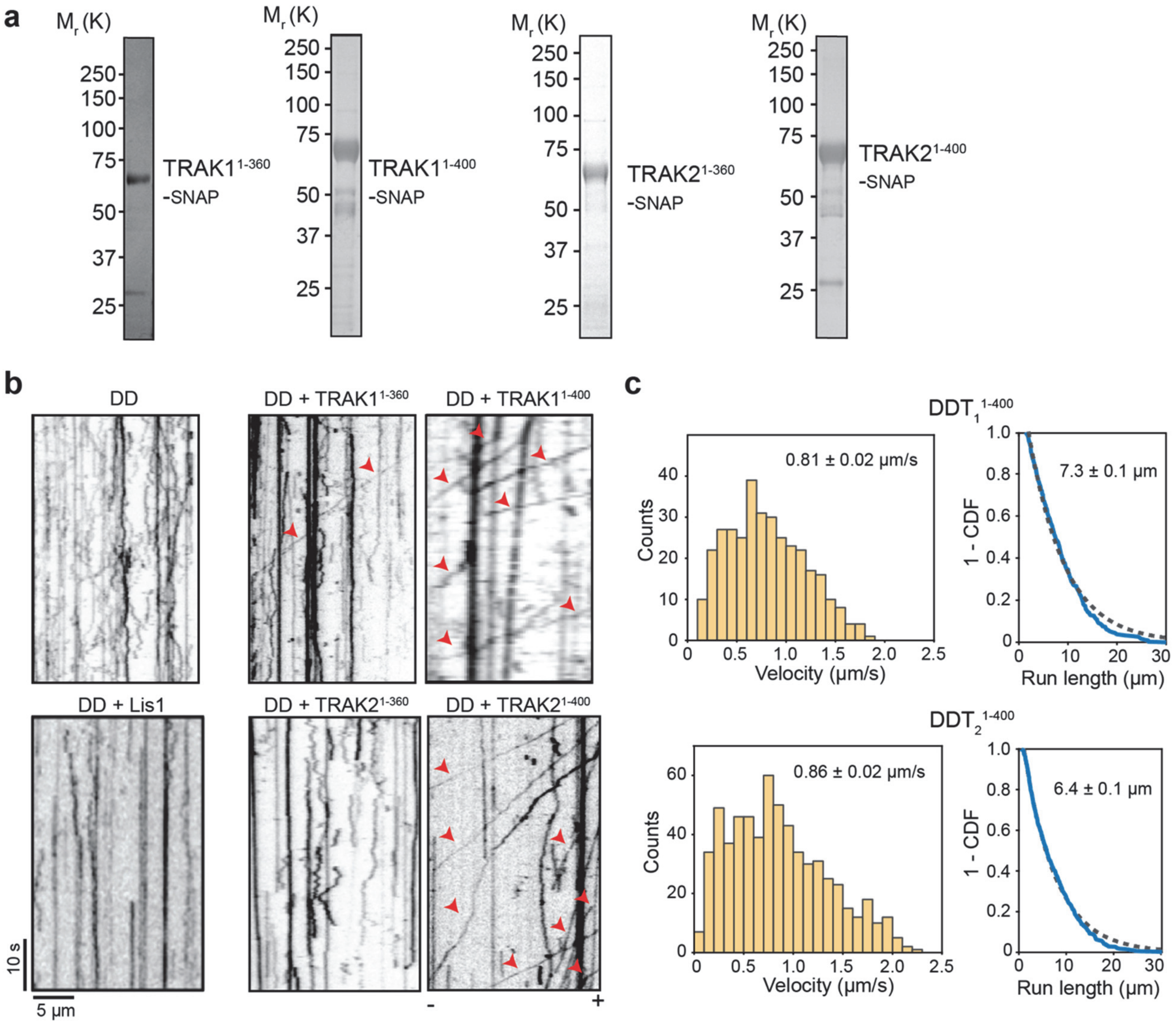

Supplementary Fig. 1 | Purification and motility of DDT complexes. a, Coomassie-stained denaturing gel of purified TRAK constructs. **b**, Representative kymographs of LD655-dynein in the presence of unlabeled dynactin and TRAK1 or TRAK2 constructs in 2 mM ATP. Arrowheads highlight processive complexes. DD and DD+Lis1 only exhibit weak microtubule binding and diffusion along the microtubule surface in the absence of a TRAK adaptor. **c**, Velocity histograms (mean ± s.e.m.) and 1-CDF of run length for $DDT_1^{1\text{-}400}$ (*n* = 341 molecules from three independent experiments) and $DDT_2^{1\text{-}400}$ (*n* = 630 molecules from three independent experiments). Fits to a single exponential decay (dashed curves) reveal the motor run lengths (±s.e.).

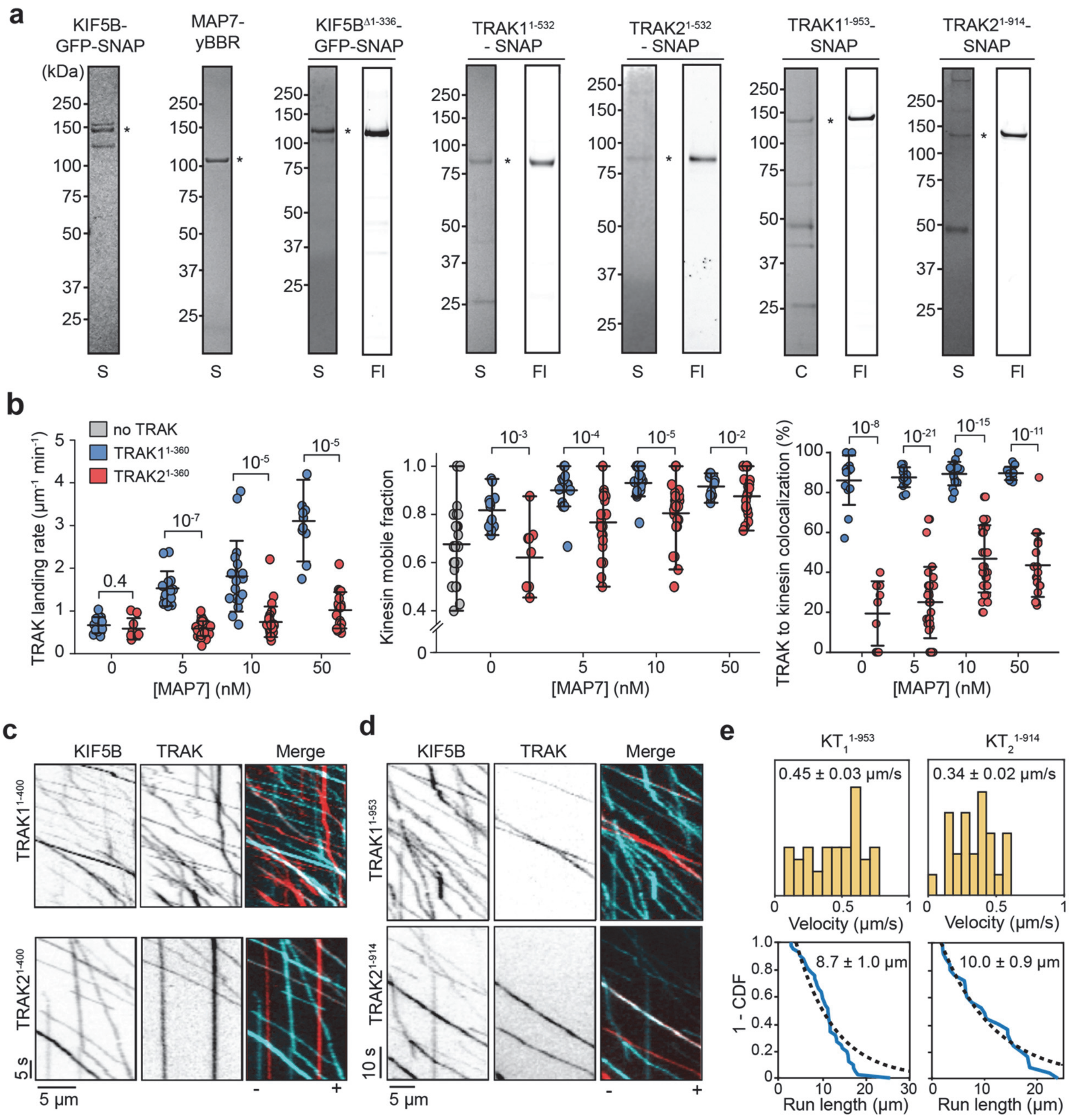

Supplementary Fig. 2 | Purification and motility of KT complexes. a, Denaturing gel pictures of purified KIF5B, MAP7, and TRAK constructs. Stars indicate the expected molecular weight (C: Coomassie-stained, S: silver-stained; Fl: fluorescence image scan). The SNAP tags of kinesin and TRAK were labeled with Alexa488 and LD555 for fluorescence imaging, respectively. **b**, (Left) The landing rate of TRAK adaptors, (Middle) the mobile fraction of kinesin motors landed on MTs, and (Right) the percentage of TRAK colocalization to processive kinesin motors moving along the MTs in the absence and presence of 5 nM TRAK1$^{1-360}$ or TRAK2$^{1-360}$ under increasing MAP7 concentrations. The center line and whiskers represent the mean and s.d., respectively (n = 26, 13, 14, 18, 12, 9, 22, 29, and 19 MTs from left to right, three independent trials). P values are

calculated from a two-tailed t-test. **c**, Representative kymographs of LD555-kinesin and LD655-labeled TRAK1$^{1\text{-}400}$ or TRAK2$^{1\text{-}400}$ in 10 nM MAP7. **d**, Representative kymographs of LD555-kinesin and LD655-labeled full-length TRAK1$^{1\text{-}953}$ or TRAK2$^{1\text{-}914}$ in 10 nM MAP7. **e,** (Top) Velocity histogram (mean ± s.e.m.) and (Bottom) 1-CDF of run length for kinesin carrying full-length TRAK1 (KT$_1$$^{1\text{-}953}$; *n* = 41, three independent experiments) or full-length TRAK2 (KT2$^{1\text{-}914}$; *n* = 25, three independent experiments). Fits to a single exponential decay (dashed curves) reveal the motor run length (±s.e.).

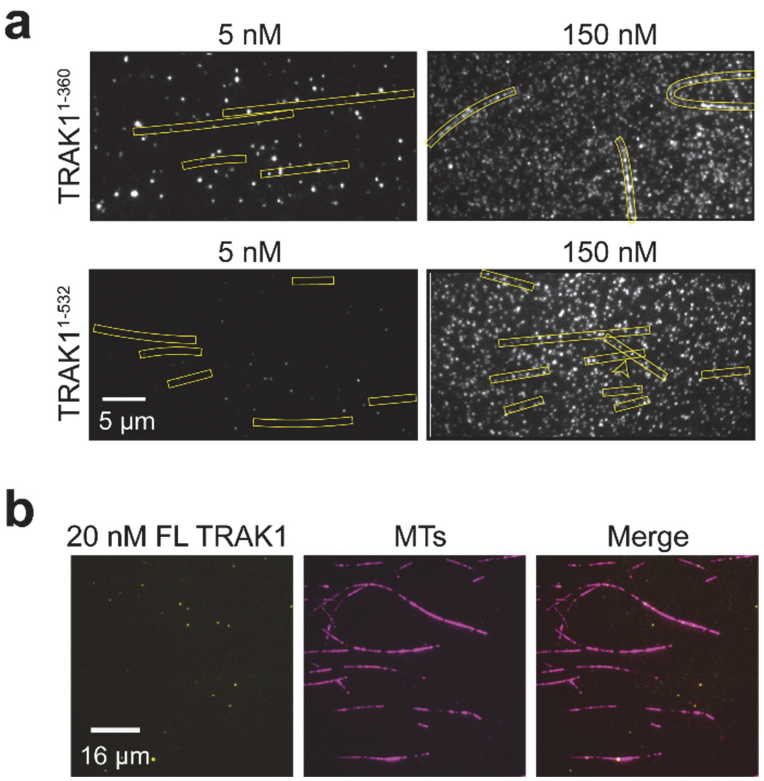

Supplementary Fig. 3 | TRAK weakly interacts with MTs. a, Sample images show localization of 5 nM and 150 nM TRAK1$^{1\text{-}360}$ or TRAK1$^{1\text{-}532}$ on surface-immobilized MTs (highlighted in yellow) in the absence of kinesin (experiments were replicated three times per condition with reproducible results). **b**, Sample images show little to no binding of 20 nM full-length TRAK1$^{1\text{-}953}$ to surface-immobilized Cy5-MTs in the absence of kinesin (experiments were replicated three times per condition with reproducible results).

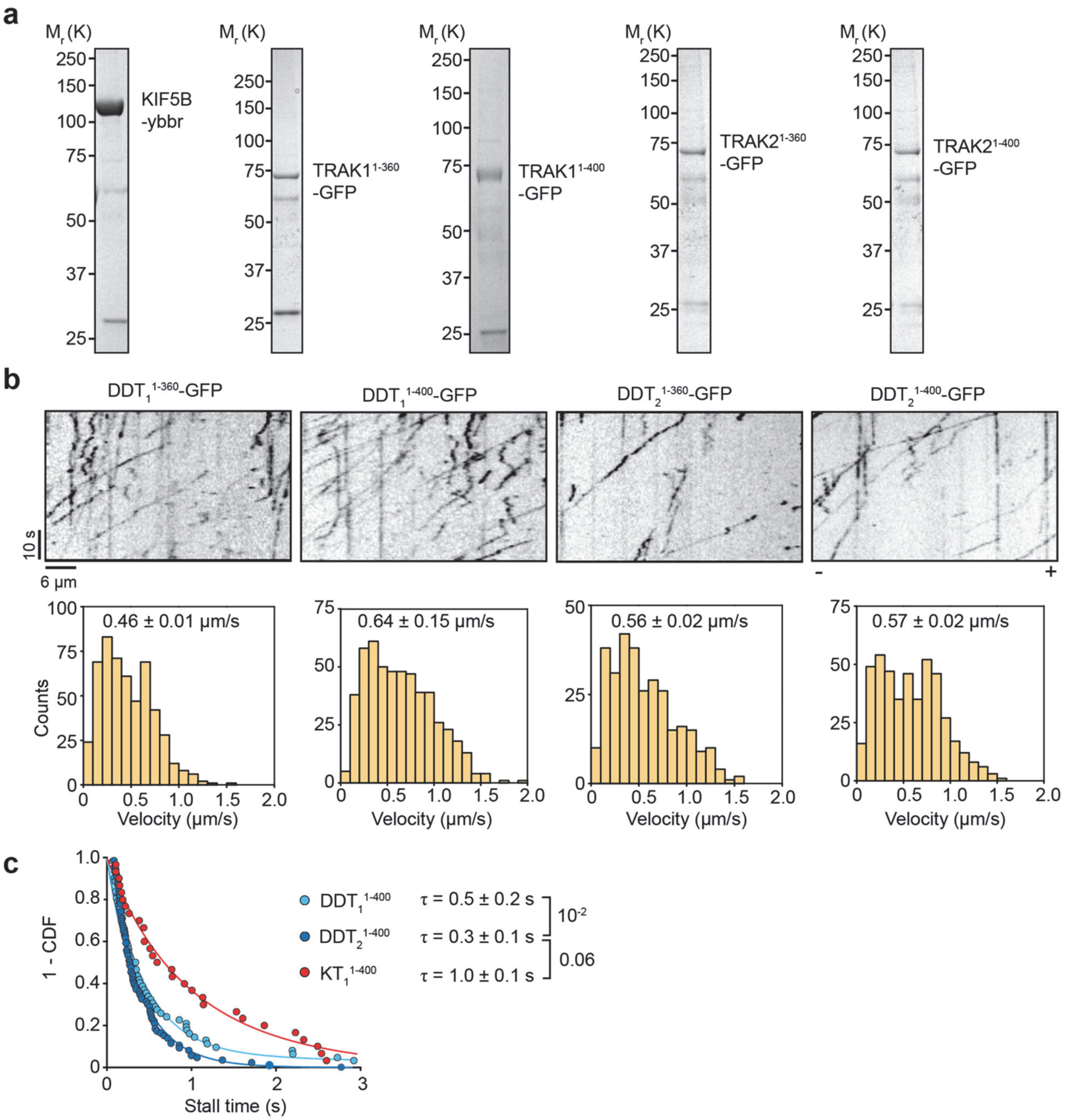

Supplementary Fig. 4 | Purification and motility of KT and DDT complexes for optical trapping experiments. a, Coomassie-stained denaturing gel of purified KIF5B-ybbR, and GFP-tagged TRAK constructs. **b**, (Top) Representative kymographs of DDT complexes in 2 mM ATP. (Bottom) Velocity histograms of DDT complexes in 2 mM ATP (mean ± s.e.m., *n* = 524, 523, 312, and 454 from left to right, three independent experiments per condition). **c**, 1-CDF of motor stall times (*n* = 62, 86, and 55 for $KT_1^{1\text{-}400}$, $DDT_2^{1\text{-}400,}$ and $DDT_1^{1\text{-}400}$, respectively). Fits to a single exponential decay (solid curves) reveal stall times (τ, ± s.e.). P-values are calculated from a two-tailed t-test.

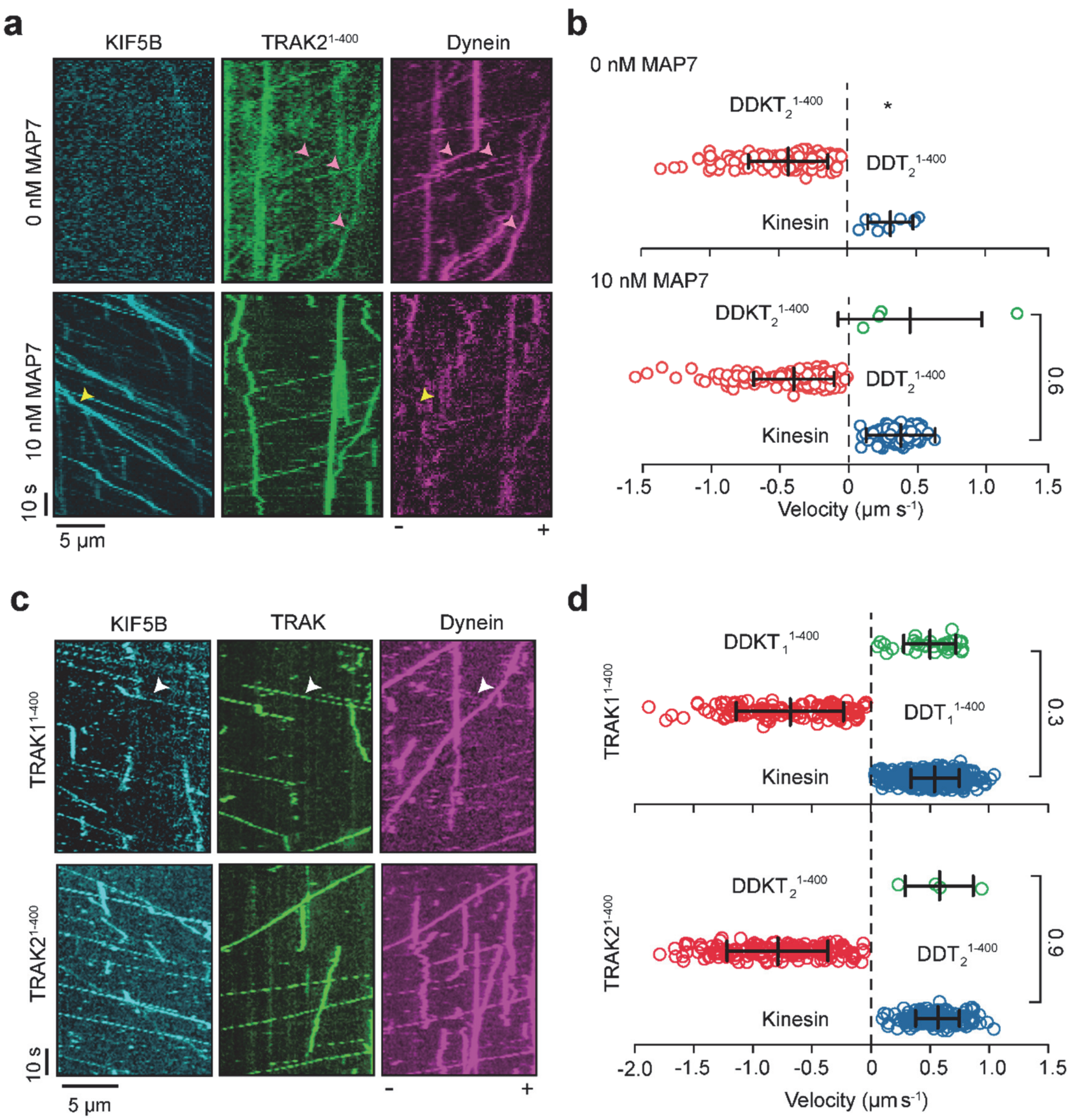

Supplementary Fig. 5 | Velocity of DDKT complexes in the presence and absence of Lis1. a, Representative kymographs of Alexa488-kinesin, LD555-TRAK2$^{1\text{-}400}$, and LD655-dynein on a surface-immobilized MT in the presence of 0 and 10 nM MAP7. Assays were performed in the absence of Lis1. Magenta arrowheads show the comigration of dynein and TRAK2, and yellow arrowheads show the comigration of dynein and kinesin towards the microtubule plus-end. **b**, The velocity distribution of individual motor complexes and DDKT assemblies at 0 and 10 nM MAP7 and 0 nM Lis1 (*n* = 0, 157, 9, 5, 180, and 234 from top to bottom, three independent experiments per condition). **c,** Representative kymographs of Alexa488-kinesin, LD555-TRAK1$^{1\text{-}400}$ or -TRAK2$^{1\text{-}400}$, and LD655-dynein on a surface-immobilized MT in the presence of unlabeled dynactin, 5 nM MAP7 and 1 μM Lis1. The white arrowhead shows the colocalization of kinesin, dynein, and TRAK. **d**, The velocity distribution of individual motor complexes and DDKT assemblies in the presence of 1 μM Lis1 (*n* = 30, 140, 439, 4, 175, and 165 from top to bottom,

three independent experiments per condition). In (**b**) and (**d**), negative velocities correspond to minus-end-directed motility. The center line and whiskers represent the mean and s.d., respectively. P-values are calculated from a two-tailed t-test.

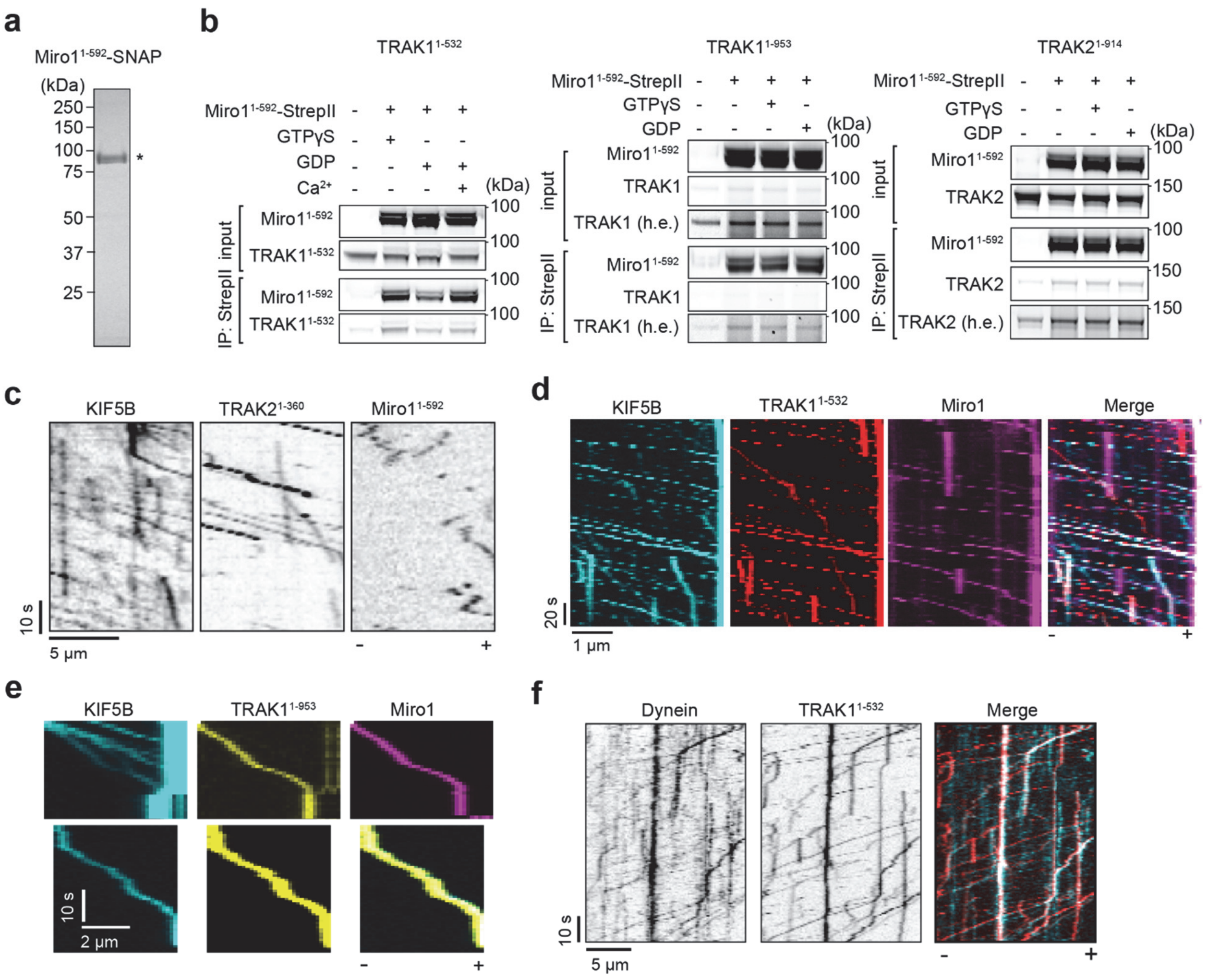

Supplementary Fig. 6 | TRAK-Miro1 interactions under different nucleotide and Ca^{2+}conditions. a, Coomassie-stained denaturing gel of purified Miro1$^{1\text{-}592}$-SNAP (experiment was repeated twice with reproducible results). **b**, In vitro immunoprecipitation of purified Miro1$^{1\text{-}592}$-SNAP-StrepII and either TRAK1$^{1\text{-}532}$ (left), full-length TRAK1 (middle), or full-length TRAK2 (right). KIF5B-GFP-SNAPf was present in all conditions. Assays were conducted in the presence of 100 µM GTPγS, 100 µM GDP, or 2 mM Ca^{2+} (h.e.: high exposure) (experiments were repeated twice with reproducible results). **c**, Representative kymograph of Alexa488-KIF5B, LD555-TRAK2$^{1\text{-}360}$, and LD655-Miro1$^{1\text{-}592}$ in the presence of 10 nM MAP7 (three independent experiments per condition). **d,** Representative kymograph of Alexa488-KIF5B, LD555-TRAK1$^{1\text{-}532}$, and LD655-Miro1$^{1\text{-}592}$ shows the formation of processive KTM complexes that walk along MTs in the presence of 10 nM MAP7 (three independent experiments per condition). **e,** Two representative kymographs of Alexa488-KIF5B, LD555-labeled full-length TRAK1, and LD655-Miro1$^{1\text{-}592}$ show the formation of processive KTM complexes that walk along MTs in the presence of 10 nM MAP7 (three independent experiments per condition). **f,** Representative kymograph of LD655-dynein and LD555-TRAK1$^{1\text{-}532}$ shows processive

motility of DDT complexes on a surface-immobilized MT in the presence of unlabeled dynactin and 1 μM Lis1 (three independent experiments per condition).

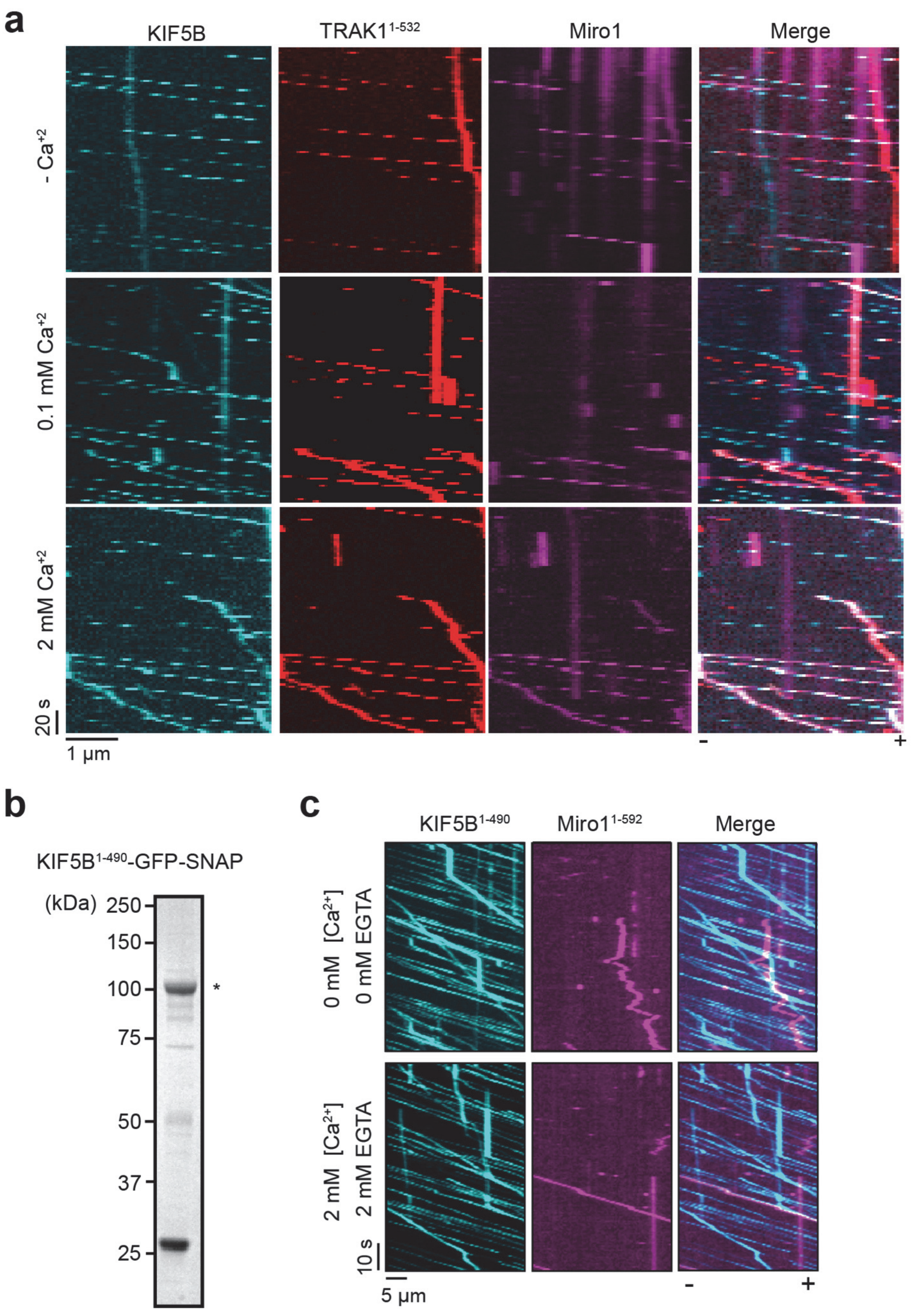

Supplementary Fig. 7 | Miro1 does not interact with the kinesin motor domain. a, Representative kymographs show comigration of Alexa488-kinesin, LD555- TRAK1$^{1\text{-}532}$, and LD655-Miro1$^{1\text{-}592}$ in 0, 0.1, and 2 mM Ca^{2+}. The assay was conducted in the presence of 10 nM MAP7 (three independent experiments per condition). **b**, Coomassie-stained denaturing gel picture of purified KIF5B$^{1\text{-}490}$-GFP-SNAP (experiment was repeated twice with reproducible results). **c**, Representative two-color kymographs of KIF5B$^{1\text{-}490}$ and Miro1$^{1\text{-}592}$ in the presence and absence of 2 mM Ca^{2+} and 2 mM EGTA. Assays were performed in the absence of TRAK and MAP7 (three independent experiments per condition).

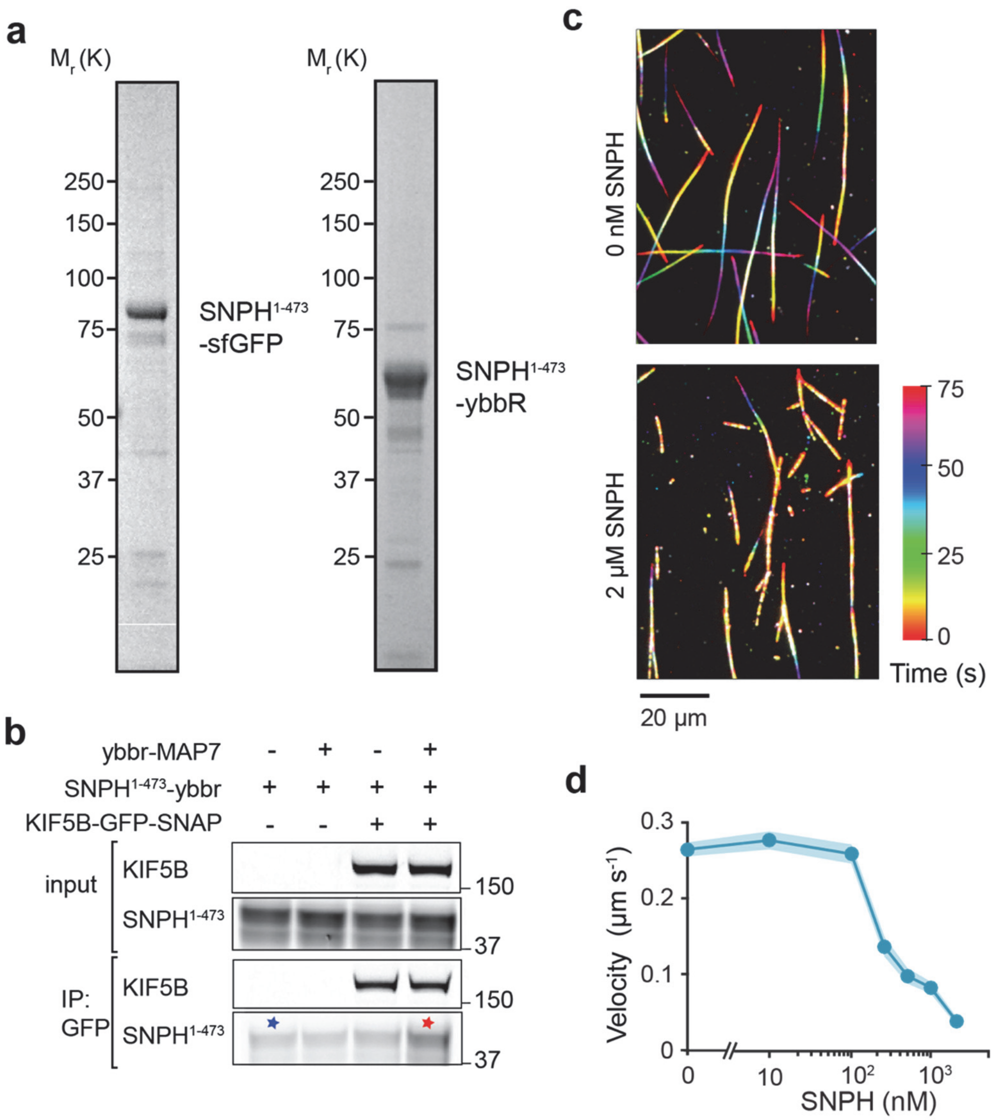

Supplementary Fig. 8 | Effect of SNPH on the MT gliding by constitutively-active kinesin. a, Coomassie-stained denaturing gel pictures of purified SNPH$^{1\text{-}473}$-sfGFP and SNPH$^{1\text{-}473}$-ybbR. **b**, In vitro immunoprecipitation shows no interaction between purified kinesin (KIF5B) and SNPH$^{1\text{-}473}$ above background (blue star) and weak interaction in the presence of MAP7 (red star). **c**, Representative color-coded projections of MT gliding driven by 2.5 nM constitutively-active KIF5B$^{1\text{-}560}$-GFP motors in the presence or absence of 2 μM SNPH-sfGFP. **d**, MT gliding velocity driven by KIF5B$^{1\text{-}560}$ motors in the presence of different SNPH$^{1\text{-}473}$-sfGFP concentrations (mean ± s.e.m., *n* = 31, 53, 67, 51, 71, 52, 68 MTs from left to right, three independent trials).
