# Supplementary material for: TRAK adaptors regulate the recruitment and activation of dynein and kinesin in mitochondrial transport
Source: Nat Commun. 2023 Mar 13;14:1376. doi: 10.1038/s41467-023-36945-8 (PMC10011603; doi:10.1038/s41467-023-36945-8)
Supplement: Supplementary file 2 — Description of Additional Supplementary Files [file 41467_2023_36945_MOESM2_ESM.pdf]

Description of Additional Supplementary Files

Supplementary Video 1: Processive motility of individual DDT complexes on MTs. One-color imaging of DDT complexes assembled with unlabeled dynein and dynactin, and LD555-labeled TRAK adaptors on surface-immobilized MTs in the presence or absence of 1 µM Lis1. Images were acquired at 250 ms per frame.

Supplementary Video 2: Processive motility of individual KT complexes on MTs. Two-color imaging of LD655-kinesin (cyan) and LD555-TRAK adaptors (red) on surface-immobilized MTs in the presence or absence of 50 nM MAP7. Images were acquired at 250 ms exposure time per frame.

Supplementary Video 3: Live imaging of DDKT1$^{1-4\circ\circ}$ complex motility on MTs. Three-color imaging of Alexa488-kinesin (KIF5B, cyan), LD555-TRAK1$^{1-400}$ (green), and LD655-dynein (magenta) on surface-immobilized MTs in the presence of unlabeled dynactin and 5 nM MAP7. Plus-end-directed motility of dynein-dynactin when it colocalizes with kinesin is highlighted by white arrows. Images were acquired at 250 ms exposure time per frame.

Supplementary Video 4: Motility of KTM complexes assembled with TRAK1 adaptors is unaffected by Ca^{2+}. Three-color imaging of Alexa488-kinesin (KIF5B, cyan), LD555-TRAK1 adaptors (red), and LD655-Miro1$^{1-592}$ (magenta) on surface-immobilized MTs with (right) or without (left and middle) 2 mM Ca^{2+}. Assays were conducted in the presence of 10 nM MAP7. Images were acquired at 250 ms exposure time per frame.

Supplementary Video 5: Motility of KTM complexes was not observed when TRAK1 was replaced with TRAK2. Three-color imaging of Alexa488-kinesin (KIF5B, cyan), LD555-TRAK2 adaptors (red), and LD655-Miro1$^{1-592}$ (magenta) on surface-immobilized MTs with (right) or without (left and middle) 2 mM Ca^{2+}. Assays were conducted in the presence of 10 nM MAP7. Images were acquired at 250 ms exposure time per frame.

Supplementary Video 6: SNPH reduces the MT landing rate and velocity of kinesin. Motility of LD655-kinesin (cyan) motility on surface-immobilized MTs in the presence or absence of 500 nM GFP-SNPH (red). SNPH decorates the MT surface. The assay was performed in 10 nM MAP7. Images were acquired at 250 ms exposure time per frame.

Supplementary Video 7: SNPH inhibits MT gliding by kinesin. Gliding motility of Cy5-MTs on surfaces decorated with 2.5 nM kinesin-GFP in the presence or absence of 500 nM GFP-SNPH. Images were acquired at 150 ms exposure time per frame.
