# Supplementary material for: TRAK adaptors regulate the recruitment and activation of dynein and kinesin in mitochondrial transport
Source: Nat Commun. 2023 Mar 13;14:1376. doi: 10.1038/s41467-023-36945-8 (PMC10011603; doi:10.1038/s41467-023-36945-8)
Supplement: Supplementary file 10 — Reporting Summary [file 41467_2023_36945_MOESM10_ESM.pdf]

nature portfolio

Corresponding author(s): John Canty, Ahmet Yildiz

Last updated by author(s): 01-27-23

Reporting Summary

Nature Portfolio wishes to improve the reproducibility of the work that we publish. This form provides structure for consistency and transparency in reporting. For further information on Nature Portfolio policies, see our Editorial Policies and the Editorial Policy Checklist.

Statistics

For all statistical analyses, confirm that the following items are present in the figure legend, table legend, main text, or Methods section.

n/a	Confirmed	
☐	☒	The exact sample size (*n*) for each experimental group/condition, given as a discrete number and unit of measurement
☐	☒	A statement on whether measurements were taken from distinct samples or whether the same sample was measured repeatedly
☐	☒	The statistical test(s) used AND whether they are one- or two-sided<br>*Only common tests should be described solely by name; describe more complex techniques in the Methods section.*
☐	☒	A description of all covariates tested
☐	☒	A description of any assumptions or corrections, such as tests of normality and adjustment for multiple comparisons
☐	☒	A full description of the statistical parameters including central tendency (e.g. means) or other basic estimates (e.g. regression coefficient) AND variation (e.g. standard deviation) or associated estimates of uncertainty (e.g. confidence intervals)
☐	☒	For null hypothesis testing, the test statistic (e.g. *F*, *t*, *r*) with confidence intervals, effect sizes, degrees of freedom and *P* value noted<br>*Give P values as exact values whenever suitable.*
☒	☐	For Bayesian analysis, information on the choice of priors and Markov chain Monte Carlo settings
☒	☐	For hierarchical and complex designs, identification of the appropriate level for tests and full reporting of outcomes
☒	☐	Estimates of effect sizes (e.g. Cohen's *d*, Pearson's *r*), indicating how they were calculated

Our web collection on statistics for biologists contains articles on many of the points above.

Software and code

Policy information about availability of computer code

Data collection	Labview 2017 was used for hardware control of the optical trap instrument. Micro-Manager was used for image acquisition.
Data analysis	Fiji 1.0 was used to analyze TIRF images. Matlab 2019a was used to process and analyze optical trap data. Figures were plotted using Fiji 1.0, Matlab 2019a, and Origin Pro 9.0. Clustal Omega from EMBL-EBI (https://www.ebi.ac.uk/Tools/msa/clustalo/) was used to align cargo adaptor sequences. The NPS@ server (https://npsa-prabi.ibcp.fr/) was used for coiled-coil domain predictions.

For manuscripts utilizing custom algorithms or software that are central to the research but not yet described in published literature, software must be made available to editors and reviewers. We strongly encourage code deposition in a community repository (e.g. GitHub). See the Nature Portfolio guidelines for submitting code & software for further information.

Data

Policy information about availability of data

All manuscripts must include a data availability statement. This statement should provide the following information, where applicable:

- Accession codes, unique identifiers, or web links for publicly available datasets
- A description of any restrictions on data availability
- For clinical datasets or third party data, please ensure that the statement adheres to our policy

A reporting summary for this article is available as Supplementary Information file. The main data supporting the findings of this study are available within the article and its Supplementary Figures. The source data underlying Figs. 1-7, Supplementary Figs. 1, 2, 4-6 and 8 are provided as a Source Data file. Additional details

on datasets and protocols that support the findings of this study will be made available by the corresponding authors upon reasonable request. Source data are provided with this paper.

Human research participants

Policy information about studies involving human research participants and Sex and Gender in Research.

Reporting on sex and gender	N/A
Population characteristics	N/A
Recruitment	N/A
Ethics oversight	N/A

Note that full information on the approval of the study protocol must also be provided in the manuscript.

Field-specific reporting

Please select the one below that is the best fit for your research. If you are not sure, read the appropriate sections before making your selection.

☒ Life sciences ☐ Behavioural & social sciences ☐ Ecological, evolutionary & environmental sciences

For a reference copy of the document with all sections, see nature.com/documents/nr-reporting-summary-flat.pdf

Life sciences study design

All studies must disclose on these points even when the disclosure is negative.

Sample size	Sample sizes were picked based on common practice in the field. In each experiment (single imaging slide) and random field of view is chosen and the number of molecules or microtubules in the field of view determines the sample size per measurement. If feasible during the experiment, multiple random fields of view per imaging slide are imaged to increase sample size and reduce the standard of the mean to at least 1/4th of the mean value.
Data exclusions	For single molecule motility assays, diffusive molecules and molecules with short run lengths (< 2 pixels or < 200 nm) or paused for more than 1 sec were excluded from the velocity analysis.<br>In optical trap assays, attachments that are shorter than 100 ms were excluded from the analysis.
Replication	Experiments were performed over the course of several months. All experiments were successfully replicated and each representative image / kymograph 'n' value represents the number of biologically distinct samples (molecules or microtubules). The number of experiments performed are given in the figure legends.
Randomization	All biological samples including proteins, microtubules, and additional assay components were aliquoted into small volumes and randomly allocated into different experimental groups. For experiments, random field of views were selected during imaging and either all events in the field of view or all events on a random microtubule were analyzed.
Blinding	Experiments were inherently blinded since it is impossible to select subpopulations of molecules, microtubules, or assay components from stock solutions when pipetting.

Reporting for specific materials, systems and methods

We require information from authors about some types of materials, experimental systems and methods used in many studies. Here, indicate whether each material, system or method listed is relevant to your study. If you are not sure if a list item applies to your research, read the appropriate section before selecting a response.

Materials & experimental systems

n/a	Involved in the study	
☐	☒	Antibodies
☐	☒	Eukaryotic cell lines
☒	☐	Palaeontology and archaeology
☒	☐	Animals and other organisms
☒	☐	Clinical data
☒	☐	Dual use research of concern

Methods

n/a	Involved in the study	
☒	☐	ChIP-seq
☒	☐	Flow cytometry
☒	☐	MRI-based neuroimaging

Antibodies

Antibodies used	Custom made anti-GFP antibodies (Covance Inc.) were purified by GFP affinity chromatography to be used for coating carboxyl latex beads (Life Technologies) and coating glass assay chambers. Commercial anti-GFP nanobodies functionalized to magnetic agarose beads (Chromotek, Inc.) were used for in vitro immunoprecipitation assays. Commercial Strep-tag II fusion proteins functionalized to sepharose resin were used for in vitro immunoprecipitation assays.
Validation	Custom made anti-GFP antibodies were verified by ELISA. Commercial anti-GFP nanobodies were verified by western blot. Commercial Strep-tag II fusion proteins were verified by western blot. Validation was provided by the supplier. Since the anti-GFP and Streptavidin conjugated beads were used for in-vitro co-IP experiments, validation was performed using negative control co-IP in the absence of a GFP-tagged or StrepII-tagged bait protein to establish that the antibody did not interact non-specifically with target proteins. Custom made anti-GFP antibodies (Covance Inc.) were purified by GFP affinity chromatography to be used for coating carboxyl latex beads (Life Technologies) and coating glass assay chambers. Commercial anti-GFP nanobodies functionalized to magnetic agarose beads (Chromotek, Inc.) were used for in vitro immunoprecipitation assays. Commercial Strep-tag II fusion proteins functionalized to sepharose resin were used for in vitro immunoprecipitation assays.

Eukaryotic cell lines

Policy information about cell lines and Sex and Gender in Research

Cell line source(s)	SF9 and HEK293F cell lines were used only for protein purification. Both cell lines were provided by the Cell Culture facility at University of California Berkeley.
Authentication	No cell line authentication was used.
Mycoplasma contamination	Cells were not tested for mycoplasma contamination.
Commonly misidentified lines (See ICLAC register)	No commonly misidentified cell lines were used.
